# Ferroptosis triggers mitochondrial fragmentation via Drp1 activation

**DOI:** 10.1038/s41419-024-07312-2

**Published:** 2025-01-25

**Authors:** Lohans Pedrera, Laura Prieto Clemente, Alina Dahlhaus, Sara Lotfipour Nasudivar, Sofya Tishina, Daniel Olmo González, Jenny Stroh, Fatma Isil Yapici, Randhwaj Pratap Singh, Nils Grotehans, Thomas Langer, Ana J. García-Sáez, Silvia von Karstedt

**Affiliations:** 1 CECAD Cluster of Excellence, University of Cologne, Cologne, Germany; 2 Institute for Genetics, University of Cologne, Cologne, Germany; 3 Department of Translational Genomics, Faculty of Medicine and University Hospital Cologne, University of Cologne, Cologne, Germany; 4 University of Barcelona, Barcelona, Spain; 5 Max Planck Institute for Biology of Ageing, Cologne, Germany; 6 Center for Molecular Medicine Cologne (CMMC), Faculty of Medicine and University Hospital Cologne, University of Cologne, Cologne, Germany; 7 Max Planck Institute of Biophysics, Frankfurt, Germany

**Keywords:** Cell death, Mitochondria

## Abstract

Constitutive mitochondrial dynamics ensure quality control and metabolic fitness of cells, and their dysregulation has been implicated in various human diseases. The large GTPase Dynamin-related protein 1 (Drp1) is intimately involved in mediating constitutive mitochondrial fission and has been implicated in mitochondrial cell death pathways. During ferroptosis, a recently identified type of regulated necrosis driven by excessive lipid peroxidation, mitochondrial fragmentation has been observed. Yet, how this is regulated and whether it is involved in ferroptotic cell death has remained unexplored. Here, we provide evidence that Drp1 is activated upon experimental induction of ferroptosis and promotes cell death execution and mitochondrial fragmentation. Using time-lapse microscopy, we found that ferroptosis induced mitochondrial fragmentation and loss of mitochondrial membrane potential, but not mitochondrial outer membrane permeabilization. Importantly, Drp1 accelerated ferroptotic cell death kinetics. Notably, this function was mediated by the regulation of mitochondrial dynamics, as overexpression of Mitofusin 2 phenocopied the effect of Drp1 deficiency in delaying ferroptosis cell death kinetics. Mechanistically, we found that Drp1 is phosphorylated and activated after induction of ferroptosis and that it translocates to mitochondria. Further activation at mitochondria through the phosphatase PGAM5 promoted ferroptotic cell death. Remarkably, Drp1 depletion delayed mitochondrial and plasma membrane lipid peroxidation. These data provide evidence for a functional role of Drp1 activation and mitochondrial fragmentation in the acceleration of ferroptotic cell death, with important implications for targeting mitochondrial dynamics in diseases associated with ferroptosis.

## Introduction

Ferroptosis is a caspase independent form of regulated necrosis characterized by the accumulation of iron-dependent lipid peroxides in cellular membranes [1]. Ferroptosis has been implicated in several oxidative stress-related diseases such as ischemia-reperfusion, degenerative diseases, acute renal failure, traumatic brain injury, and cancer [2–6].

Reactive oxygen species (ROS) in cells can be triggered by several causes, such as impaired redox capacity, increased mitochondrial respiration, imbalanced iron metabolism, or, in the presence of labile iron, increased Fenton reactions generating hydroxyl radicals. Under physiological conditions, ROS produced by the respiratory chain in the inner mitochondrial membrane (IMM) are buffered by cytosolic and mitochondrial antioxidant enzymes, whose inactivation represents the main trigger of ferroptosis. A key function in the defense against ferroptosis is assigned to glutathione peroxidase 4 (GPX4), which depends on the presence of glutathione (GSH) as a co-factor [1, 7]. As a result, direct inhibition of GPX4 by small molecule inhibitors such as RSL3 or ML210, or depletion of GSH levels using erastin, are widely used methods to induce ferroptosis [1, 7]. Given that intracellular cystine is needed for GSH synthesis, ferroptosis protection is thereby also critically regulated through cystine import via the cystine/glutamate antiporter xCT, which is a molecular target of erastin [8]. As a second line of defense, the oxidoreductase ferroptosis suppressor protein 1 (FSP1) was recently described to generate ubiquinol from ubiquinone, the first of which acts as a radical-trapping agent halting lipid peroxidation specifically at the plasma membrane [9, 10].

Mitochondria have been proposed to play an important role in ferroptosis. Notably, the major site of ubiquinone synthesis lies within mitochondria. On the other hand, plasma, mitochondrial and endoplasmic reticulum (ER) membranes have been suggested as potential major sites of lipid peroxidation in ferroptosis [11]. In addition, several changes in mitochondrial morphology have been linked to oxidative stress [12]. It is thus not surprising that early in the characterization of ferroptotic cell death, mitochondrial fragmentation was reported [1]. Inhibition of xCT in addition to mitochondrial fragmentation has also been shown to induce mitochondrial ROS production, loss of mitochondrial membrane potential (MMP), and ATP depletion [13–17]. In support of a requirement for mitochondrial metabolism in the execution of ferroptosis, mitochondrial depletion via Parkin-mediated mitophagy in vitro or inhibition of oxidative phosphorylation (OXPHOS) rescued cells from ferroptosis induced by cystine deprivation or erastin treatment [13]. However, mitochondrial DNA-depleted cells remained sensitive to oxidative stress and ferroptosis induction [1]. Mitochondrial fragmentation has also been observed in various types of regulated cell death besides ferroptosis [1], such as apoptosis [18, 19], necroptosis [20] and pyroptosis [21].

The large GTPase Dynamin-related protein 1 (Drp1) promotes constitutive mitochondrial fission in healthy human cells to maintain cellular homeostasis, and it was shown to mediate mitochondrial fragmentation and cristae remodeling in apoptotic cells to facilitate cytochrome c release [18, 19]. During apoptosis, Drp1 can directly interact with BAX at apoptotic foci, promoting mitochondrial outer membrane permeabilization (MOMP) and finally cell death [19]. Drp1-mediated mitochondrial fragmentation has also been observed in necroptosis [22, 23] and autophagy [24]. Notably, heterozygous *drp1* knockout (KO) mice show defective mitochondrial fission and lower levels of lipid peroxidation in tissues [25]. However, despite the massive mitochondrial fragmentation observed in ferroptotic cells, the contribution of Drp1 to ferroptotic cell death remains poorly understood.

Here, we characterized kinetics of mitochondrial alterations in cells undergoing ferroptosis in relation to lipid peroxidation. We find that Drp1 is activated upon ferroptosis and accelerates ferroptotic cell death. This function of Drp1 in ferroptotic cell death acceleration is dependent on its mitochondrial recruitment and regulation of mitochondrial dynamics. Our findings support a role for Drp1 in remodeling of the mitochondrial network and in cell death promotion during ferroptosis.

## Results

### Ferroptosis induces mitochondrial fragmentation and depolarization, but not mitochondrial outer membrane permeabilization

To first determine whether mitochondrial integrity might play a role in ferroptosis, we used live-cell confocal microscopy to explore alterations in mitochondrial morphology and function upon ferroptosis induction over time. We used the lipid peroxidation sensor BODIPY C11 581/591 to visualize lipid peroxidation in cell membranes, Mitotracker deep red as a marker of mitochondrial network morphology, and Tetramethylrhodamine ethyl ester perchlorate (TMRE) to visualize changes in mitochondrial membrane potential.

**Fig. 1:**
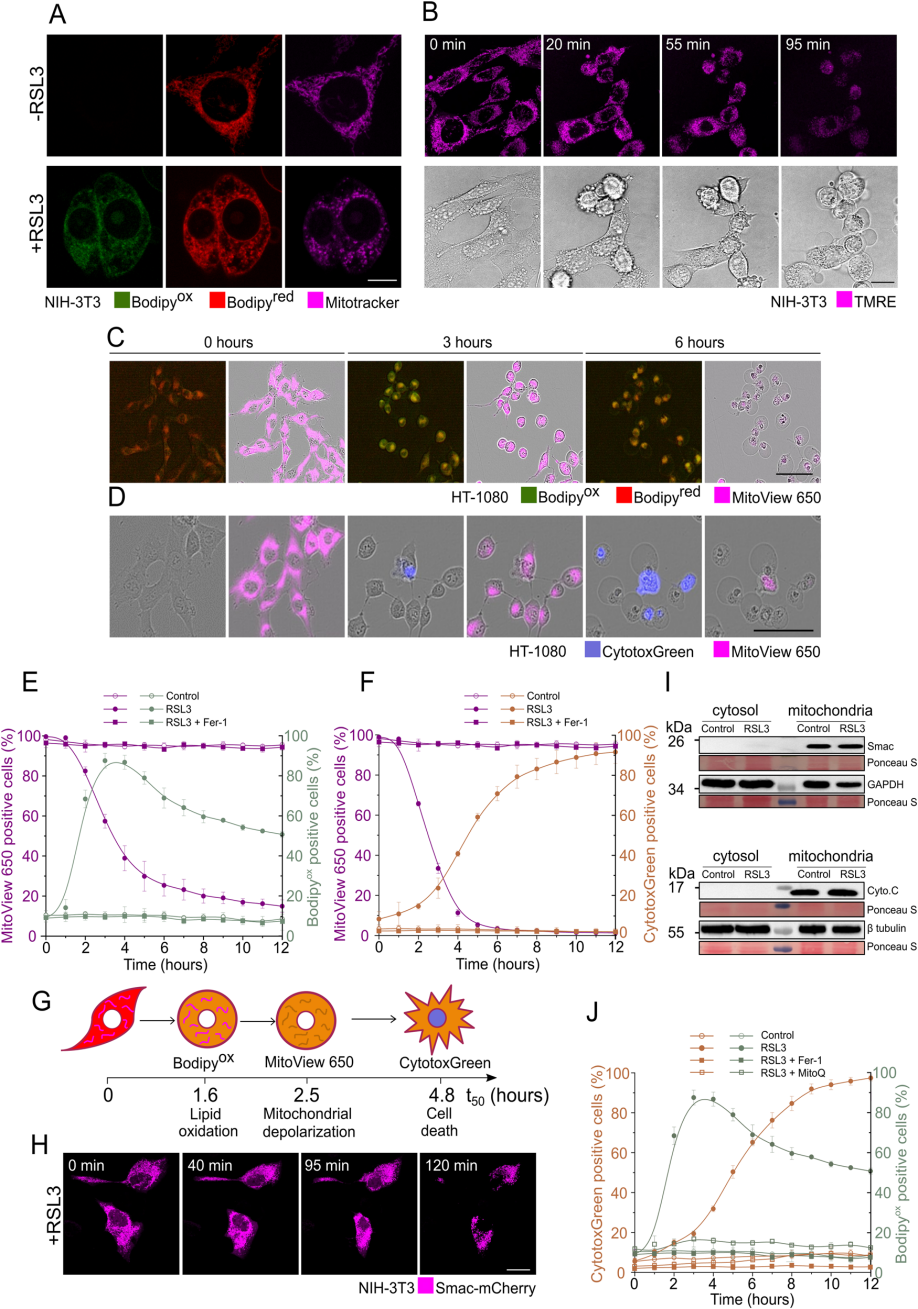
Ferroptosis induces mitochondrial fragmentation and depolarization but not mitochondrial outer membrane permeabilization. **A** Live cell confocal images of NIH-3T3 cells labeled with Bodipy C-11 [1 µM] and Mitotracker [50 nM] ± RSL3 treatment [2 µM] for 2 h. Scale bar, 10 µm. **B** Time-lapse confocal images of NIH-3T3 cells treated with RSL3 [2 µM] and monitored for loss of TMRE staining [200 nM]. Scale bar, 20 µm. **C**, **D** Time-lapse Incucyte images of HT-1080 cells treated with RSL3 [0.5 µM] and labeled using Bodipy C-11 [1 µM] and MitoView 650 [50 nM] in **c** or CytotoxGreen staining [250 nM] and MitoView 650 staining [50 nM] in (**D**). Scale bars in (**C**, **D**), 100 µm. **E** Kinetics of increase in lipid peroxidation and decrease in MitoView 650 positive cells after RSL3 [0.5 µM] treatment in the presence or absence of Fer-1 [2 µM], calculated from experiments as shown in (**C**). **F** Kinetics of increase in CytotoxGreen staining and decrease in MitoView 650 positive cells after RSL3 [0.5 µM] treatment ± Fer-1 [2 µM], calculated from experiments as shown in (**D**). **G** Graphical representation of the sequence of events observed in HT-1080 cells following RSL3-induced ferroptosis. t_50_ of each phenotypic event was calculated from the mean curves shown in (**E**, **F**). These values correspond to the time at 50% of the maximum signal. **H** Time-lapse confocal microscopy images of Smac-mCherry cellular localization during ferroptosis induction in NIH-3T3 cells treated with RSL3 [1 µM]. Scale bar, 20 µM. **I** HT-1080 cells were treated with DMSO (control) or RSL3 [1 µM] for 3 h. Cells were lyzed and separated into cytosolic and mitochondrial fractions. The indicated proteins were detected by Western blotting. **J** Time course of increase in oxidized Bodipy C-11 and CytotoxGreen uptake in HT-1080 cells treated with RSL3 [0.5 µM] in the presence or absence of MitoQ [0.5 µM] or Fer-1 [2 µM]. Images are representative of at least three independent experiments each performed at least in triplicates throughout. Values in (**E**, **F**, **J**) represent the mean of at least three independent experiments ± STDEV.

Upon induction of ferroptosis using the GPX4 small molecule inhibitor RSL3, we observed an increase in oxidized BODIPY C11 indicative of increased lipid ROS, increased fragmentation of the mitochondrial network and loss of mitochondrial membrane potential in NIH3T3 cells (Fig. 1A, B). Mitochondrial depolarization was accompanied by cell shape changes, including cell rounding and the appearance of a single swollen bleb at the plasma membrane, which is considered a ferroptotic hallmark (Fig. 1B, lower panel) [26].

To further characterize the temporal relationship between increased lipid ROS, loss of mitochondrial membrane potential and cell death, we tracked all these events in parallel in HT-1080 cells treated with RSL3 using fluorescent live cell imaging (Fig. 1C, D). We used MitoView 650 to visualize changes in mitochondrial membrane potential [27, 28] and the fluorescent DNA-intercalating agent CytotoxGreen as a marker of irreversible plasma membrane rupture and cell death. We detected a clear increase in oxidized BODIPY C11 indicative of lipid peroxidation, which preceded the loss of mitochondrial membrane potential and cell death in HT-1080 cells upon RSL3 treatment (Fig. 1C–F). All of these events were inhibited by the lipophilic radical-trapping agent and specific ferroptosis inhibitor ferrostatin-1 (Fer-1) [1], indicating their specific dependence on lipid peroxidation (Fig. 1E, F). From the kinetic curves (Fig. 1E, F), we determined the time required to reach 50% of each parameter (*t*_50%_) which allowed us to determine the lag time between these ferroptotic phenotypes (Fig. 1G). Temporally, after RSL3 treatment, HT-1080 cells showed an increase in lipid peroxidation (*t*_50%_ = 1.6 h) followed by a decrease in mitochondrial membrane potential (*t*_50%_ = 2.5 h) and cell death (*t*_50%_ = 4.8 h) (Fig. 1G). Mitochondria are major sites of iron utilization, especially for the synthesis of iron sulfur clusters [29]. To test whether mitochondrial membrane depolarization could also affect availability of cytosolic and/or mitochondrial labile iron pools, we performed live cell imaging of labile iron pools in HT-1080 cells treated with RSL3. We observed a clear increase in labile iron pools preceding cell death (Fig. S1A–D), which could be blocked with the iron chelating agent deferoxamine (DFO) (Fig. S1B). Of note, the labile iron pools were distributed throughout the cell without a preferred subcellular localization (Fig. S1D).

In apoptosis, MOMP, characterized by loss of mitochondrial membrane potential and release of cytochrome c and Smac/DIABLO into the cytosol, is considered the point of no return in the cells’ commitment to death [30]. Smac is a mitochondrial pro-apoptotic factor that is released into the cytosol upon MOMP [19]. To next determine whether the observed mitochondrial changes were accompanied by MOMP, we performed live-cell confocal imaging of RSL3-treated HT-1080 cells transiently expressing Smac-mCherry (Fig. 1H). In contrast to MOMP during apoptosis [19, 31–33], there was no release of Smac into the cytosol despite loss of mitochondrial potential in NIH-3T3 cells undergoing ferroptosis (Fig. 1H). This result was confirmed by immunoblot assay using an anti-Smac antibody in HT-1080 cells (Fig. 1I). Similarly, we did not detect the release of cytochrome c from the intermembrane space of mitochondria into the cytosol of HT-1080 cells upon ferroptosis induction (Fig. 1I), indicating that loss of mitochondrial membrane potential is not followed by MOMP in ferroptotic cell death.

The contribution of oxidation of subcellular organelles to ferroptosis execution is not yet clear [13]. Mitochondria generate a high amount of ROS in the respiratory chain that are potentially able to oxidize polyunsaturated fatty acid (PUFA)-containing phospholipids through non-enzymatic reactions [13]. Studies on the role of mitochondria in ferroptosis suggested that mitochondria play an important role in cysteine starvation-induced ferroptosis by competitively consuming GSH as a cofactor for GPX4 to reduce lipid peroxides generated in mitochondria and plasma membrane [13]. However, how mitochondria-derived ROS contribute to the execution of ferroptotic cell death remains an open question. To shed light on this issue, we characterized the effect of Mitoquinol (mitoQ), a mitochondria-targeted ubiquinol analog [34] and radical trapping agent, on ferroptotic death in HT-1080 cells treated with RSL3. Both lipid peroxidation and cell death were prevented in the presence of MitoQ, suggesting that ROS residing within mitochondria are relevant to ferroptosis execution.

### Drp1 accelerates ferroptotic cell death

**Fig. 2:**
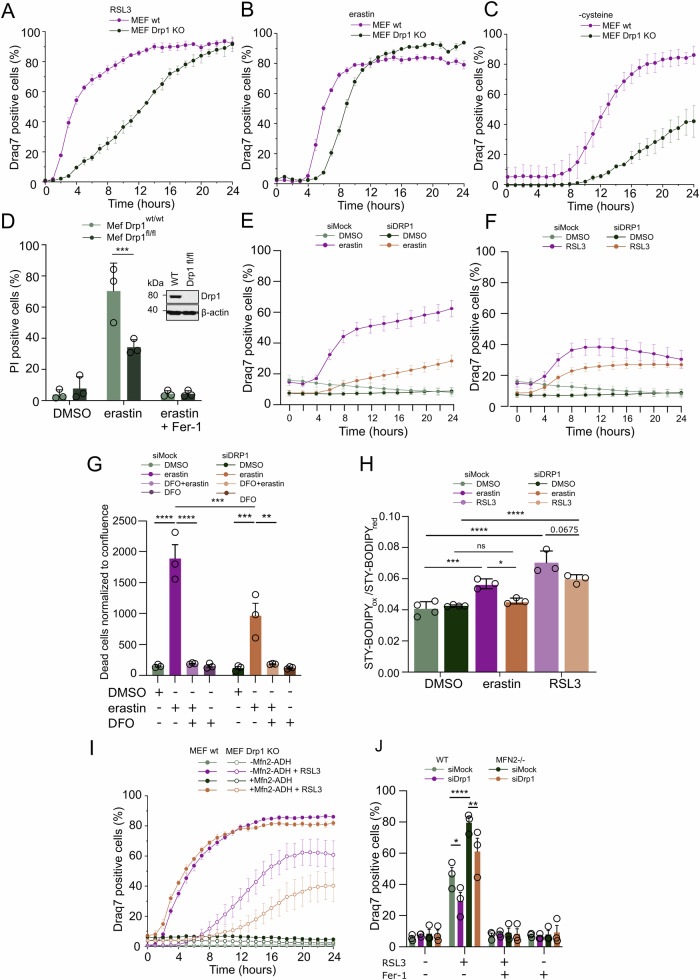
Drp1 accelerates ferroptotic cell death. Control or *drp1*^*−/−*^MEFs were treated with **A** RSL3 [0.5 µM], **B** erastin [10 µM] or **C** cysteine starvation in the presence of Draq7 [3 µM] for the indicated time. Dead cells were quantified as Draq7 positive cells using Incucyte live cell imaging. **D** Control or *drp1*^*FL/FL*^ MEFs cells were infected with Adenoviral-Cre for 24 h. The indicated cells were subsequently treated with DMSO or erastin [10 µM] for 16 h. % Propidium iodide (PI) positive cells were quantified by flow cytometry. Representative immunoblots of the used cells (insert). Wild-type MEFs were subjected to mock or Drp1-targeting siRNA for 72 h followed by treatment with **E** erastin [10 µM] or **F** RSL3 [1 µM]. Dead cells were quantified as Draq7 positive cells using Incucyte live cell imaging. **G** wild-type MEFs ± Drp1 knockdown as in (**E**, **F**) were treated with erastin [10 µM] and/or DFO [10 µM]. Dead cells were quantified as Draq7 positive cells normalized to cell confluence using Incucyte live cell imaging. **H** wild-type MEFs ± Drp1 knockdown as in (**E**, **F**) were treated with erastin [10 µM] or RSL3 [1 µM] in the presence of STY-BODIPY [1 µM] for 4 h and imaged using the Incucyte live cell imaging system. The ratio of oxidized STY-Bodipy (STY-Bodipy_ox_) to reduced STY-Bodipy (STY-Bodipy_red_) is shown. **I** Control or Drp1 knockout (KO) MEFs were transiently transfected with mitofusin 2-ADH (Mfn2-ADH) for 24 h and subsequently treated with RSL3 [0.5 µM] in the presence of Draq7 [3 µM] for the indicated time and imaged using the Incucyte live cell imaging. **J** wild-type or Mitofusin-2 Knockout (*mfn2*^*−/−*^) MEFs were subjected to mock or Drp1-targeting siRNA for 72 h followed by treatment with RSL3 [1 µM] and/ or Fer-1 [1 µM]. Dead cells were quantified as Draq7 positive cells using Incucyte live cell imaging. All graphs represent means ± STDEV or ±SEM of at least three independent experiments each performed at least in triplicates throughout. Ordinary one-way ANOVA and multiple comparisons test. ****p < 0.0001, ***p < 0.001, **p < 0.01, *p < 0.05.

In mammals, mitochondrial fission and fragmentation are mediated by Drp1, which translocates from the cytosol to the outer mitochondrial membrane (OMM), where it oligomerizes into spirals that are believed to mediate mitochondrial constriction and subsequent mitochondrial fragmentation [15, 18, 35]. Drp1-mediated mitochondrial fragmentation has been described in various types of programmed cell death, including necroptosis [20, 36], autophagy [24, 37] and apoptosis [18, 19]. However, the contribution of Drp1 to ferroptosis is only poorly understood. To investigate whether Drp1 plays a role in the mitochondrial fragmentation observed in ferroptotic cells (Fig. 1A) and in ferroptotic cell death, we assessed the effect of Drp1-deficiency in mouse embryonic fibroblasts (MEFs) treated with RSL3, erastin or cysteine starvation (Fig. 2A–C). We observed that regardless of ferroptosis-inducing treatment (Fig. S2A–C), there was a delay in the kinetic of cell death in absence of Drp1, especially at early time points, suggesting that Drp1 presence accelerates ferroptosis kinetics (Fig. 2A–C). Similarly, transient genetic depletion of *drp1* using *drp1*^fl/fl^ MEFs infected with adenoviral Cre efficiently reduced induction of ferroptosis (Fig. 2D). In addition, siRNA-mediated silencing of Drp1 in MEFs and in human H441 and A549 non-small cell lung cancer (NSCLC) cell lines reproduced this effect when undergoing ferroptosis induced by RSL3, erastin or buthionine sulfoximine—an irreversible inhibitor of γ-glutamylcysteine synthetase that causes GSH depletion (Fig. 2E, F, Fig. S2D–I). Notably, the iron chelating agent DFO blocked residual cell death irrespective of the presence or absence of Drp1 indicating that iron was required in both cases (Fig. 2G). Moreover, levels of mitochondrial iron transport proteins were not changed by Drp1 silencing (Fig. S2J). We also observed a decrease in cellular lipid peroxidation after erastin or RSL3 treatment upon Drp1 silencing (Fig. 2H). Accordingly, treatment with the Drp1 inhibitor Mdivi also partially protected H441 and A549 cells from ferroptotic cell death (Fig. S2K).

To counterbalance Drp1-mediated mitochondrial fission, the large GTPases Mitofusin 1 and 2 (Mfn1/2) promote mitochondrial fusion [38, 39]. As such, both Drp1-deficiency and Mfn1/2 overexpression results in more fused and elongated mitochondria [40]. Yet, Drp1 has also been described to fulfill other functions independent of mitochondrial dynamics [38]. To assess whether the ferroptosis-promoting effect of Drp1 is related to its role in regulating mitochondrial dynamics, we transiently transfected wild type (WT) and *drp1*^*−/−*^ MEFs with alcohol dehydrogenase (ADH)-tagged Mfn2-ADH (Fig. S2L) and measured the kinetic of RSL3-induced ferroptosis. Interestingly, in *drp1*^*−/−*^ MEFs, overexpression of Mfn2 further protected cells from ferroptosis (Fig. 2I). The protective effect of Mfn2 overexpression against ferroptosis was not reproduced in WT MEFs, probably because we were unable to achieve significant levels of expression under similar conditions (Fig. S2L). In support of a function for mitochondrial dynamics in regulating ferroptosis kinetics, *Mfn2*^*−/−*^ MEFs were more sensitive to RSL3-induced ferroptosis than controls, which could be reverted upon Drp1 silencing (Fig. 2J, Fig. S2M). Collectively, these data suggest that it is the regulation of mitochondrial dynamics, rather than an independent specific function of Drp1, that modulates ferroptosis execution.

### Drp1 is activated and translocates to mitochondria upon ferroptosis induction

**Fig. 3:**
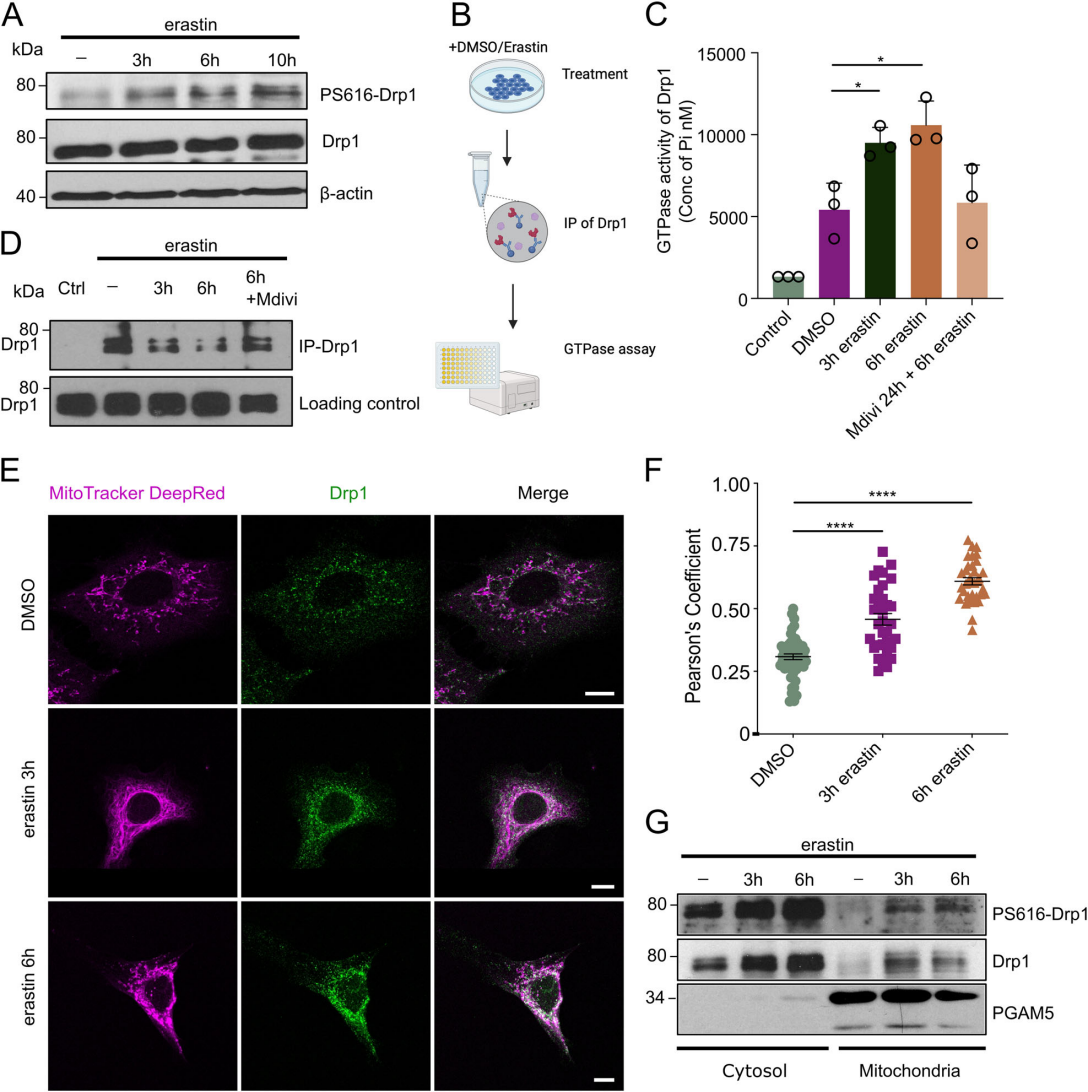
Drp1 is activated and recruited to mitochondria upon induction of ferroptosis. **A** A549 cells were treated with erastin [10 µM] for the indicated time, lyzed and subjected to Western blotting. **B** schematic illustration of experimental setup in (**C**, **D)**. **C**, **D** A549 cells were pre-treated with Mdivi-1 [75 µM] for 24 h followed by additional treatment with erastin [10 µM] for the indicated time points followed by immunoprecipitation of Drp1 and **C** GTPase activity assay with one part and **D** Western blotting of another part. **E** Confocal images of A549 labeled with Mitotracker [150 nM] and co-stained with Drp1-Antibody, ±erastin treatment [10 µM] for the indicated time points. Scale bar, 10 µm. **F** quantification of colocalization of Drp1 and mitochondria using Pearson’s coefficient. Images were analyzed using ImageJ and Jacob Plugin. Pearson’s coefficient values are plotted. Every dot represents a single cell quantified. **G** representative immunoblots of isolated mitochondria and their respective cytosolic fraction of A549 cells after treatment with erastin [10 µM] for the indicated time points. Images are representatives of at least three independent experiments. Data represent means from three independent experiments ±STDEV. Ordinary one-way ANOVA + multiple comparisons test. ****p < 0.0001, ***p < 0.001, **p < 0.01, *p < 0.05.

Drp1 is a cytosolic protein that requires translocation to the OMM to induce fission events [41, 42]. This translocation depends on mitochondrial adapter proteins and phosphorylation events of Drp1 [43, 44]. The most common and important phosphorylation sites are serine 616 (S616) and serine 637 (S637), which have opposite effects on Drp1 regulation [45]. Phosphorylation at S616 enhances Drp1 activity and promotes its translocation to mitochondria, whereas phosphorylation at S637 suppresses Drp1 activity [35, 46]. Interestingly, we observed a time-dependent phosphorylation of Drp1 S616 in cells treated with erastin (Fig. 3A) suggesting that cytosolic Drp1 is activated upon ferroptosis induction.

The GTPase activity of Drp1 is a central factor in the induction of oligomerization at the OMM and ultimately, in mitochondrial fission. Therefore, we tested whether Drp1 GTPase activity is increased after ferroptosis induction by isolating Drp1 by immunoprecipitation followed by GTPase activity assays (Fig. 3B). We found that the GTPase activity of Drp1 indeed increased over time after erastin treatment (Fig. 3C, D). Accordingly, treatment of cells with the Drp1 inhibitor Mdivi-1 reverted the increased GTPase activity of Drp1 during erastin-induced ferroptosis (Fig. 3C, D). Both the increased phosphorylation at Ser616 and GTPase activity of Drp1, suggested that Drp1 might translocate to mitochondria during ferroptosis. Indeed, using confocal microscopy, we observed significant recruitment of Drp1 to mitochondria following erastin treatment (Fig. 3E, F). Moreover, mitochondrial fractions isolated from cells treated with DMSO or erastin, also showed a time-dependent increase in S616-phosphorylated and total Drp1 (Fig. 3G). In summary, we find that Drp1 is activated and accumulates at mitochondria upon induction of ferroptosis.

### Mitochondrial recruitment of Drp1 is required for promotion of ferroptosis

**Fig. 4:**
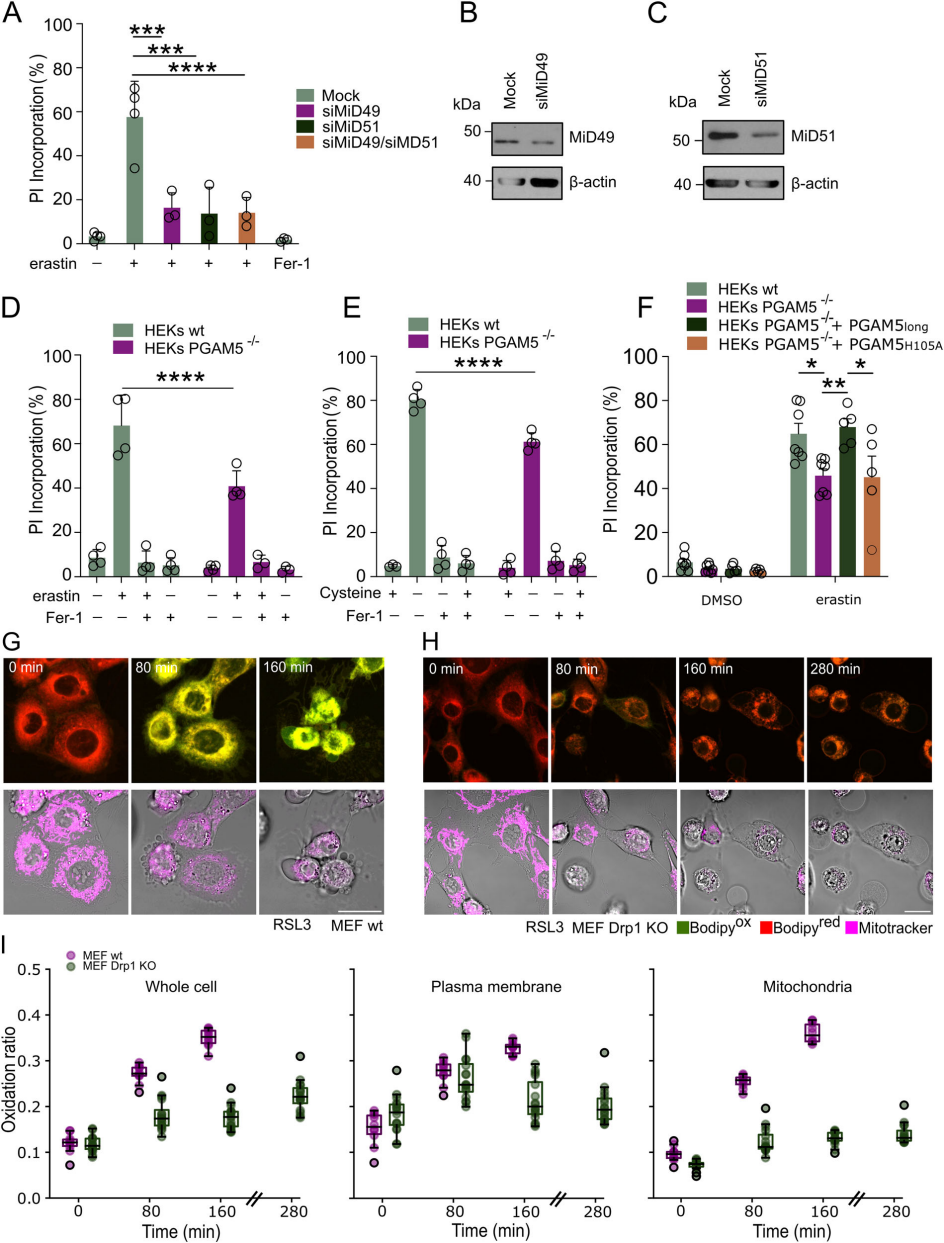
Mitochondrial Drp1 recruitment accelerates ferroptotic cell death. **A** A549 cells were subjected to mock or MiD49- and/or MiD51-targeting siRNAs for 72 h followed by erastin treatment [10 µM] for 24 h. Cell death was quantified by propidium iodide (PI) uptake and flow cytometry. **B**, **C** Representative Western blots are shown. Wild type and PGAM5^−/−^ HEK cells were subjected to (**D**) erastin [10 µM] or (**E**) cysteine starvation ± Fer-1 treatment [1 µM] for 24 h. Cell death was determined as in (**A**). **F** Wild type and PGAM5^−/−^ HEK cells or PGAM5^−/−^ HEK cells containing a doxycycline-inducible PGAM5-expression plasmid (WT or H105A) were subjected to doxycycline treatment [100 nM] for 24 h followed by erastin treatment [10 µM] for 24 h and cell death analysis using PI staining. **G** Time lapse live cell confocal images of wt and **H** *drp1*^*−/−*^ MEFs labeled with BODIPY-C11 [1 µM] and Mitotracker Deep Red [150 nM], and treated with RSL3 [4 µM]. Scale bar, 20 µm. The upper panel shows merged color channels: oxidized BODIPY-C11 in green and non-oxidized BODIPY-C11 in red. The lower panel displays Mitotracker Deep red fluorescence and bright field images of the equatorial focus plane. **I** Quantification of the oxidation ratio (Bodipy^ox^/(Bodipy^ox ^+ Bodipy^red^)) in wt (*n* = 10 cells) and *drp1*^*−/−*^ MEFs (*n* = 16 cells), scatter box plot displays the interquartile range (IQR) and median oxidation ratio. Points beyond 1.5 times IQR are considered outliers. Values in (**a**, **d**–**f**) represent the mean of three independent experiments each performed at least in duplicates ± STDEV. Statistical test: Two-way ANOVA + Tukey’s multiple comparison test. ****p < 0.0001, ***p < 0.001, **p < 0.01, *p < 0.05.

Drp1 recruitment to mitochondria is initiated by its phosphorylation, but also by the presence of adapter proteins that facilitate its localization to the OMM [43]. In mammals, the OMM proteins mitochondrial fission factor (Mff), mitochondrial fission 1 protein (Fis1), mitochondrial dynamics of 49 kDa (MiD49) and mitochondrial dynamics of 51 kDa (MiD51) are described as adapters of Drp1 [43]. However, while Fis1 and Mff are also localized in other organelles such as peroxisomes, MiD49 and MiD51 are exclusively found in mitochondria [47, 48]. Drp1-dependent mitochondrial fission events driven by MiD49 and MiD51 were shown to be required for cytochrome c release during the early phase of intrinsic apoptosis [18]. To test whether these mitochondrial adapters are also involved in ferroptotic cell death, we used siRNA-mediated knockdown of MiD49 or MiD51 upon erastin treatment followed by a cell death read-out using propidium iodide (PI) staining. Remarkably, partial silencing of these mitochondrial adapters was sufficient to rescue cells from undergoing ferroptosis (Fig. 4A–C).

While phosphorylation of Drp1 at S616 activates it, dephosphorylation of the inhibitory S637 site is important for the oligomerization of Drp1 and therefore for the execution of fission events [49]. During intrinsic and extrinsic apoptosis, Drp1 is recruited to fission sites on mitochondria where it is further activated in a secondary step by S637-Drp1 dephosphorylation by the mitochondrial phosphoglycerate mutase/protein phosphatase (PGAM5) [50]. PGAM5 functions as a Ser/Thr phosphatase and a proportion of it has been shown to localize to the OMM with its C-terminus facing the cytoplasm [51]. To determine/confirm PGAM5 localization in our cellular system, we isolated mitochondria from cells treated with erastin for several time points and subjected them to proteinase K-mediated digestion of OMM-exposed proteins. While the bona-fide OMM protein translocase of outer mitochondrial membrane protein 70 (TOM70) was effectively cleaved off the mitochondrial surface, PGAM5 behaved similar to the IMM control protein translocase of the inner membrane protein 23 (TIM23), i.e., a sub-proportion was constitutively cleaved off while a larger proportion was inaccessible for proteinase K-mediated cleavage (Fig. S3A). Importantly, inducing ferroptosis using erastin did not change proteinase K-accessibility over time indicating that a proportion of PGAM5 is indeed constitutively available at the OMM, but that this amount does not change upon induction of ferroptosis.

Recently, PGAM5 was found to interact with Mfn2 and Drp1 in a stress-sensitive manner [52]. We thus tested whether PGAM5 might be involved in ferroptosis. Interestingly, we found that *PGAM5*^*−/−*^ cells were indeed more resistant to erastin and cysteine-deprivation-induced ferroptosis compared to WT cells (Fig. 4D–E). Moreover, reconstitution of *PGAM5*^*−/−*^ cells with cDNAs of the long isoform of WT (Fig. S3B) but not catalytically inactive (H105A) PGAM5 [53] restored ferroptosis sensitivity to wild-type cell levels (Fig. 4F) indicating that expression and catalytic activity of this isoform is sufficient to promote ferroptosis.

Since Drp1 depletion slowed down ferroptosis kinetics, we hypothesized that the subcellular spread of lipid peroxidation from intracellular membranes to the plasma membrane [11] might be impaired in absence of Drp1. To address this, we developed a method in which we stained mitochondria using Mitotracker, which we used to generate a binary mask to determine mitochondrial co-localization with oxidized BODIPY C11 staining in 3D stacks. Plasma membrane oxidation was estimated in parallel using the contour of the cells in bright field images as a reference. This approach allowed single cell quantification of subcellular lipid peroxidation distribution in real-time. Strikingly, we found that, in contrast to wild-type cells, lipid peroxidation at the plasma membrane was initially unchanged but impaired after 80 min in *drp1*^*−/−*^ cells. Importantly, mitochondrial lipid peroxidation accumulation was strongly impaired in *drp1*^*−/−*^ cells throughout (Fig. 4G–I). Taken together, these data suggest that mitochondrial fragmentation mediated by Drp1 promotes mitochondrial lipid peroxidation and subcellular lipid peroxidation spread thereby accelerating ferroptosis kinetics.

## Discussion

While rapid progress has been made in our understanding of ferroptosis in recent years, the relevance of mitochondrial alterations to ferroptosis progression and the underlying molecular mechanisms involved have remained poorly understood. Here, we investigated several mitochondrial alterations during ferroptosis execution including an increase in mitochondrial fragmentation and mitochondrial depolarization, and kinetically correlated them with lipid oxidation. We found that GPX4 inactivation induces an increase in lipid peroxidation that precedes mitochondrial depolarization and complete plasma membrane disruption.

Mitochondrial fragmentation is a feature of ferroptosis [1, 13, 14, 54] that is common to a variety of programmed cell death pathways such as apoptosis [18, 19, 30], necroptosis [20] and pyroptosis [21]. Early studies in the ferroptotic field have proposed that mitochondria are fragmented due to the accumulation of ROS [1]. However, whether peroxidation of PUFAs-PL at mitochondria would be sufficient to cause mitochondrial fragmentation has not been investigated. In apoptosis, mitochondrial fragmentation is mainly triggered by the GTPase Drp1 and has been associated with MOMP, which releases cytochrome c and Smac/DIABLO into the cytosol [18, 30, 55]. However, mitochondrial fragmentation is conserved in apoptotic cell death, even in organisms where MOMP is not involved [56]. Interestingly, we find that mitochondrial fragmentation and potential loss are also not associated with MOMP in ferroptosis.

Interfering with the mitochondrial fission machinery by depletion or silencing of Drp1 delays ferroptosis execution, unraveling an important role of this protein in ferroptosis. Our results also showed that overexpression of Mfn2 in Drp1 KO MEFs partially rescues ferroptosis. Interestingly, homozygous *drp1* KO mice are embryonically lethal [57, 58] whereas heterozygous *drp1* KO mice survive and show lower levels of H_2_O_2_ and lipid peroxides in tissues compared to wild-type littermates [25]. In line with the ferroptosis-promoting function for Drp1, Drp1 activity was correlated with tissue ferroptosis levels in injury models of temporary middle cerebral artery occlusion/reperfusion [59]. Consistent with this, we observed decreased lipid peroxidation in response to ferroptosis induction in the mitochondria and plasma membrane of Drp1-deficient MEFs. However, Drp1 has previously been suggested to limit the induction of ferroptosis using erastin in oral squamous cell carcinoma [60]. Since erastin can also induce generic ROS-driven cell death due to the resulting loss of GSH and that the latter study used the general ROS scavenger N-acetyl cysteine to revert cell death, it is possible that Drp1 can fulfill additional and opposing functions depending on whether lipid ROS or general ROS are the driver of cell death.

The activity of Drp1 is dependent on several post-translational regulations, of which phosphorylation at S616 and S637 are the best described. P-S616 induces Drp1 activation and translocation to mitochondria, but some studies have shown that P-S616 does not necessarily affect Drp1 GTPase activity [56, 57]. In this study, we found that Drp1 is phosphorylated at S616 and translocates to mitochondria upon ferroptosis induction hand in hand with an increase in GTPase activity. In necroptosis, PGAM5 has been described to recruit Drp1 and activate its GTPase activity by dephosphorylating P-S637 [22, 61]. Consistent with this, we also observed decreased cell death in PGAM5^−/−^ cells compared to the corresponding control. PGAM5 activation during mitochondrial stress has been specifically described in response to interfering with the electron transport chain, subsequently triggering mitochondrial fission and mitophagy [22]. It has been proposed that PGAM5 interacts with and dephosphorylates FUN14 Domain Containing 1 (FUNDC1) at serine 13 in response to mitochondrial depolarization or hypoxia, thereby enabling the interaction between the LC3-interacting region (LIR) of FUNDC1 and LC3, which represents a key step in mitophagy [62]. Additionally, BCL2L1, but not BCL2 interacts with and inhibits PGAM5, to prevent the dephosphorylating of FUNDC1, which activates hypoxia-induced mitophagy [63]. PGAM5 also dephosphorylates Bcl-xL at Ser 62, restoring BCL-xL inhibition of BAX and BAK and resistance to apoptosis [64]. Conversely, oxidative stress drives the oligomerization of PGAM5, causing its dissociation from BCL-xL, consequently promoting apoptosis. In this regard, it has been proposed that the interaction of PGAM5 with FUNDC1 and BCL-xL, which is dependent on PGAM5’s oligomerization state, serves as a pivotal point in the switch between mitophagy and apoptosis [64]. In our conditions, it is reasonable to speculate that PGAM5 is activated during oxidative stress during ferroptosis, leading to mitochondrial fragmentation, although the molecular mechanism is still unknown. Interestingly, a recent study reported protein-protein interactions between Drp1 and ACSL4 and a requirement for Drp1 dephosphorylation at S637 to stabilize ACSL4 and promote ferroptosis [65]. Hence, it is possible that the observed failure to accumulate peroxidized lipids in mitochondria observed in Drp1-deificient cells is promoted by alterations within the organelle-specific lipidome.

In summary, our results support a general role for Drp1 in controlling mitochondrial alterations during the progression of different types of regulated cell death.

## Materials and methods

### Reagents

erastin (Bectin Pharma, Calbiochem®, Germany), Mdivi-1 (Sigma Aldrich), Ferrostatin-1 (Sigma Aldrich), RSL3 (Sellekchem), zVAD (Enzo Life Sciences), Nec1s (Abcam), DFO (Merck). RPMI without methionine, cysteine and L-glutamine was supplemented with 100 nM methionine and 2 mM L-glutamine, 10% FCS and 1000 U/mL of both penicillin and streptomycin (all from Sigma Aldrich). DMEM medium was purchased from (Invitrogen, Germany). C11 BODIPY 581/591, Draq7 and CytotoxGreen were purchased from Thermofisher (Germany).

### Generation of cell lines

HEK293 Flp-In T-Rex PGAM5 KO cells were generated using CRISPR-SpCas9 mediated gene editing. Cells were transiently transfected with the px335 (#42335) expression plasmid containing hSpCas9n (D10A) nickase and hPGAM5 guide RNAs. Cell cultures were subjected to monoclone selection by serial dilution. Single clones were validated by immunoblotting and genomic sequencing. PGAM5 KO cells were complemented by stable integration of C-terminally Myc-labeled murine PGAM5 (long isoform) or PGAM5 H105A at the FRT site followed by immunoblot verification.

### Cell lines and culture conditions

Human NSCLC cell lines (H441, A549) were kindly provided by Prof. Julian Downward. They were cultured in RPMI 1640 medium (Thermo Fisher) supplemented with 10% fetal bovine serum (FBS) (Sigma Aldrich) and 1000 U/mL of both penicillin and streptomycin (Sigma Aldrich). Mfn2^−/−^ Murine embryonic fibroblasts (MEFs) were kindly provided by Prof. Lena Pernas (University of California Los Angeles, Los Angeles). NIH-3T3 cells were kindly provided by Prof. Dr. Andreas Linkermann (University Hospital Carl Gustav Carus of the Technical University of Dresden). HT-1080 cells were provided by Dr. Marcus Conrad (Helmholtz Zentrum München) under a material transfer agreement. Drp1^fl/fl^ MEFs were kindly provided by Luca Scorrano (Veneto Institute of Molecular Medicine, Padova). NIH-3T3, HT-1080, HEK, and wt and Drp1 KO MEFs were cultured in Dulbecco´s modified Eagle´s medium (DMEM, Thermo Fisher) supplemented with 10% FBS (Sigma Aldrich), 2 mM L-glutamine and 1000 U/mL of both penicillin and streptomycin (Sigma Aldrich). All cells were cultured at 37 °C in humidified atmosphere containing 5% CO_2_ and periodically tested for mycoplasma (Mycoplasma barcodes, Eurofins genomics). Cells were frequently passaged at subconfluence and seeded at a density of 0.5–5 × 10^4^ cells/mL.

### Reverse transfection with siRNA

For Drp1 knockdown experiments, 300,000 NSCLC cells or 150,000 MEFs were plated on top of mixed Dharmafect Reagent I (Dharmacon) and the specific siRNA (stock 20 mM, SMART-pool, Dharmacon) in 6-well plates for 72 h.

### IncuCyte assay

The kinetics of cell death, lipid peroxidation, labile iron pools and mitochondrial membrane potential were recorded using the IncuCyte bioimaging platform (Essen, Germany). Cells were seeded in 96-well plates (10^4^ cells per well) 1 day prior to treatment. After treatment, four images per well were acquired every hour, analyzed and averaged using *cell-by-cell* analysis software provided by the manufacturer. Cell death was measured by incorporation of Draq7, PI or CytotoxGreen. The labile iron pools were visualized using Bio Tracker TM 575 Red Fe^2+^. All dyes were used at the concentrations recommended by manufactures. Data were collected as the number of cell positive for each fluorescent marker and divided by the total number of cells detected in the bright field under each condition. The oxidation of C-11 Bodipy 581/591 or STY-BODIPY was calculated as an indicator of lipid peroxidation per cell and was estimated based on the fluorescence intensity per pixel of the green channel fluorescence images corresponding to the oxidized fraction of Bodipy (Bodipy^ox^). *t*_50%_ of Bodipy^ox^, cell death, labile iron pools and mitochondrial depolarization were calculated by fitting the corresponding kinetic curves to a single exponential growth function using Origin 8.0.

### Bright field and confocal microscopy imaging

Cells were seeded in DMEM in IBIDI eight-well chambers (Ibidi, Germany) 24 h before the experiment. The next day, the cells were washed with PBS and the media was replaced with phenol red-free DMEM (Sigma-Aldrich, Germany) supplemented with FBS and antibiotics. Cells were loaded with the appropriate fluorescent dye for 30 min at 37 °C. Confocal microscopy was performed using a gSTED Leica confocal microscope (Leica Microsystems) equipped with a 63X, NA = 1.4 oil immersion CS2 objective on the sample. Excitation light came from Argon ion (488 nm) or HeNe (561 nm) lasers. Fluorescence emission was detected using HyD SMD detectors. Live cell imaging was performed under 5% (v/v) CO_2_ and temperature control at 37 °C. For Drp1 translocation to mitochondria studies, compounds were added 1 day after plating and cells were incubated for 3, 6 or 24 h. For mitochondrial staining, MitoTracker Deep Red (150 nM) was added to each well during the last 30 min of incubation. After staining, cells were washed twice in PBS, fixed with 4% formaldehyde for 20 min, permeabilized with 0.1% Triton X-100 in PBS for 5 min, and blocked with 0.1% Triton X-100, 1% BSA in PBS for 1–2 h at room temperature. Cells were incubated with primary Drp1 antibody (CST, 8570) at 4 °C overnight, washed three times with PBS, followed by incubation with the appropriate secondary antibody at 37 °C for 1–2 h at room temperature. Finally, cells were washed five times in PBS and stored at 4 °C in 100 µL of PBS per well until imaging. Images were adjusted for brightness and contrast using Fiji/ImageJ. Fiji´s Jacob plug-in from was used to quantify co-localization. For visualizing iron labile pools cells were loaded with Bio Tracker TM 575 Red Fe^2+^ (4 µM) 30 min after ferroptosis induction. To measure the subcellular distribution of oxidized lipids we stained mitochondria with Mitotracker DeepRed (150 nm) and Bodipy C-11 (1 µM) for 30 min at 37 °C. We then generated a binary mask from the Mitotracker signal obtained to determine mitochondrial co-localization with oxidized BODIPY C11 staining in 3D stacks, while plasma membrane oxidation was estimated using as a reference the contour of the cells in the transmitted light images. All images were processed in Fiji.

### PI uptake/Cell death FACS assay

To determine cell death, 25,000 cells were plated in 500 µL of media in each well of a 24-well plate. Compounds were added 1 day after plating and cells were incubated for 48 h followed by staining with PI (1 µg/ml) (Sigma Aldrich) in PBS (Thermo Fisher) supplemented with 2% FBS. PI-positive cells were quantified by flow cytometry using an LSR-FACS Fortessa (BD Bioscience) and FlowJo software (BD Bioscience). Flow cytometry data were collected from a minimum of 5000 cells with at least three replicates per condition.

### Western blotting

After treatment, cells were washed in PBS, lysed in IP lysis buffer (30 mM Tris-HCl pH 7.4, 120 mM NaCl, 2 mM EDTA, 2 mM KCl, 1% Triton X-100, 1× complete protease inhibitor cocktail) and frozen at -20°C. After thawing, lysate concentrations were adjusted to equal protein concentrations using the bicinchoninic acid (BCA) protein assay (Biorad). Equal amounts of protein were mixed with a final concentration of 1x reducing sample buffer (Invitrogen) and 200 mM DTT (VWR). Samples were heated to 80 °C for 10 min, separated by gel electrophoresis, and transferred to nitrocellulose membranes (Biorad) using the TurboBlotting system (Biorad). Membranes were blocked for at least 30 min in PBS containing 0.1% Tween 20 (PBST) (VWR) with 5% (w/v) dried milk powder (AppliChem). The membranes were then incubated overnight at 4 °C with primary antibodies against DRP1 (Cell signaling, 14647), PS616-DRP1 (CST, 3455), PS637-DRP1 (CST, 4867) PGAM5 (Sigma, HPA036978), MiD49 (Sigma Aldrich, PA5-46624), MiD51 (Sigma Aldrich, PA5-43348), ß-actin (Sigma Aldrich, A1978), α‐Cytc (BD, 556433), α‐GAPDH (Cell signaling, 97166S), β-tubulin (Cell signaling, 55559), Smac/Diablo (Cell signaling, D5S3R), α‐TOM70 (Santa Cruz, sc390545), α‐TIM23 (BD, BD611223), α‐Mfn2 (Abcam, ab56889) α‐Mitoferrin-1 (Proteintech, 26469-1-AP), α‐Frataxin (Abcam, ab113691), all diluted 1:1000 in PBST containing 5% bovine serum albumin (BSA) (Thermo Fisher). After washing with PBST, the membranes were incubated with horseradish peroxidase (HRP)-coupled secondary antibodies (Biotium) diluted 1:10,000 for at least 1 h at room temperature. After another wash, bound antibodies were detected using chemiluminescent Classico Western HRP Substrate (Millipore) and x-ray films (Thermo Fisher) or Imaging System Fusion Solos S (Vilber) with the Software Fusion Solo 7S Edge.

### Transduction with AdenoCre virus

For the inducible drp1 KOs, 250 µL Opti-MEM and 0.75 µL Polybrene Transfection Reagent were mixed and used per well of a 6-well plate. The mixture was incubated at room temperature for 5–10 min. Next, 2 × 108 UI of AdenoCre virus (Vectors Uniowa, Ad4364) was added to the mixture. The transduction solution was added to each well of a 6-well plate containing 100,000 inducible drp1-floxed MEFs in 1 mL media. The 6-well plates were centrifuged at 2500 rpm at 30 °C for 45 h. The transduction mixture was incubated for 24 h. After incubation, the cells were washed 3 times in PBS 1x and incubated in fresh media for another 24 before adding the specific treatment.

### Immunoprecipitation of Drp1

First, endogenous Drp1 was isolated from A549 cells instead of using recombinant Drp1. Cells were treated with the indicated compounds for the indicated time before isolation of total protein. Protein was extracted as described for Western blotting. 50 µL of protein G agarose beads were taken per condition and incubated with 0.01 µg/µL Drp1 antibody (CST, 8570) or 1 µg isotype control antibody (CST, 3900) at 4 °C overnight. After incubation, the Drp1/isotype-protein-G-agarose beads mixture was centrifuged and washed three times in RIPA lysis buffer. Whole cell protein from each group was allowed to bind to the Drp1-protein G-agarose beads mixture overnight at 4 °C.

### GTPase activity assay

After incubation with the protein-antibody mixture (Drp1), samples were centrifuged and washed five times with RIPA lysis and extraction buffer and twice with GTPase buffer (50 µM Tris-HCl pH 7.5, 2.5 µM MgCl2) for 30 min at 30 °C. Isolated Drp1 was incubated with GTP for 30 min at 30 °C. The released free phosphate was quantified using a high throughput colorimetric GTPase assay kit according to the manufacturer´s protocol. Optical density (OD) at 620 nm was measured with a plate reader.

### Mitochondrial isolation

Cytosolic and mitochondrial fractions were isolated from cultured A549 cells. Briefly, 1.8 × 10^6^ cells were plated on 10 cm petri dishes and treated with DMSO or erastin (10 µM) for 3 and 6 h, respectively. After treatment, cells were centrifuged, and the pellets were dried and frozen at −80 °C. Mitochondrial and cytosol fractions were isolated using a mitochondrial/cytosol fractionation kit according to the manufacturer´s protocol (Abcam, ab110171). For mitochondrial OMM protein digestion the mitochondrial pellet was resuspended in isotonic buffer (10 mM HEPES-KOH, pH 7.4, 0.22 M mannitol, 0.07 M sucrose) and incubated for 10 min. Subsequently, 0.5 μg/mL of proteinase K was added and incubated for 10 min. The reaction was stopped by adding Laemmli buffer, followed by boiling at 85 °C for 10 min prior to loading the samples onto SDS-PAGE gels for protein analysis.

### Cytochrome c and Smac release assay

To measure the release of cytochrome c and Smac from mitochondria, 5 × 10^5^ HT-1080 cells were treated with RSL3 (1 µM) for 3 h to induce ferroptosis. For separation of mitochondria and cytosol, cells were harvested by trypsinization, washed in PBS and permeabilized with permeabilization buffer (20 mM HEPES/KOH pH7.5, 100 mM sucrose, 2.5 mM MgCl_2_, 100 mM KCl, freshly added 0.025% (w/v) digitonin and protease inhibitor cocktail in PBS) for 10 min on ice. Cell membranes were pelleted by centrifugation at 15,000 × *g* for 10 min at 4 °C. After removal of the supernatant (cytosolic fraction), the membranes were solubilized with RIPA buffer. Protein levels were analyzed by Western blot.

### Quantification and statistical analysis

Statistical analysis was performed using GraphPad software (GraphPad Software Inc.). Two-tailed *t*-tests were used to compare two conditions, and two-way ANOVA and Bonferroni post-test were used to compare multiple samples. All measurements were performed at least three times, and results are presented as mean ± standard deviation.

## Supplementary information

Supplementary Material

Original Data File

## Data Availability

All original data are available from the corresponding authors upon reasonable request.
