# Supplementary material for: Ferroptosis triggers mitochondrial fragmentation via Drp1 activation
Source: Cell Death Dis. 2025 Jan 25;16(1):40. doi: 10.1038/s41419-024-07312-2 (PMC11762985; doi:10.1038/s41419-024-07312-2)
Supplement: Supplementary file 1 — Supplementary Material [file 41419_2024_7312_MOESM1_ESM.pdf]

Supplementary Data

Ferroptosis triggers mitochondrial fragmentation via Drp1 activation

Lohans Pedrera[1,2,*], Laura Prieto Clemente[1, 3,*], Alina Dahlhaus[1, 3,*], Sara Lotfipour Nasudivar[1,2], Sofya Tishina[1,3], Daniel Olmo González[1,3,4], Jenny Stroh[1,3], Fatma Isil Yapici[1,3], Randhwaj Pratap Singh[1,2], Nils Grotehans[5], Thomas Langer[1,5,6], Ana J. García-Sáez[1,2,7, #] & Silvia von Karstedt[1,3,6, #]

[1]CECAD Cluster of Excellence, University of Cologne, Cologne, Germany.

[2]Institute for Genetics, University of Cologne, Cologne, Germany

[3]Department of Translational Genomics, Faculty of Medicine and University Hospital Cologne, University of Cologne, Cologne, Germany.

[4]University of Barcelona, Barcelona, Spain.

[5]Max Planck Institute for Biology of Ageing, Cologne, Germany

[6]Center for Molecular Medicine Cologne (CMMC), University of Cologne, Faculty of Medicine and University Hospital Cologne, Germany.

[7]Max Planck Institute of Biophysics, Frankfurt, Germany.

[*]These authors contribute equally.

[#] Co-corresponding authors.

Figure S1

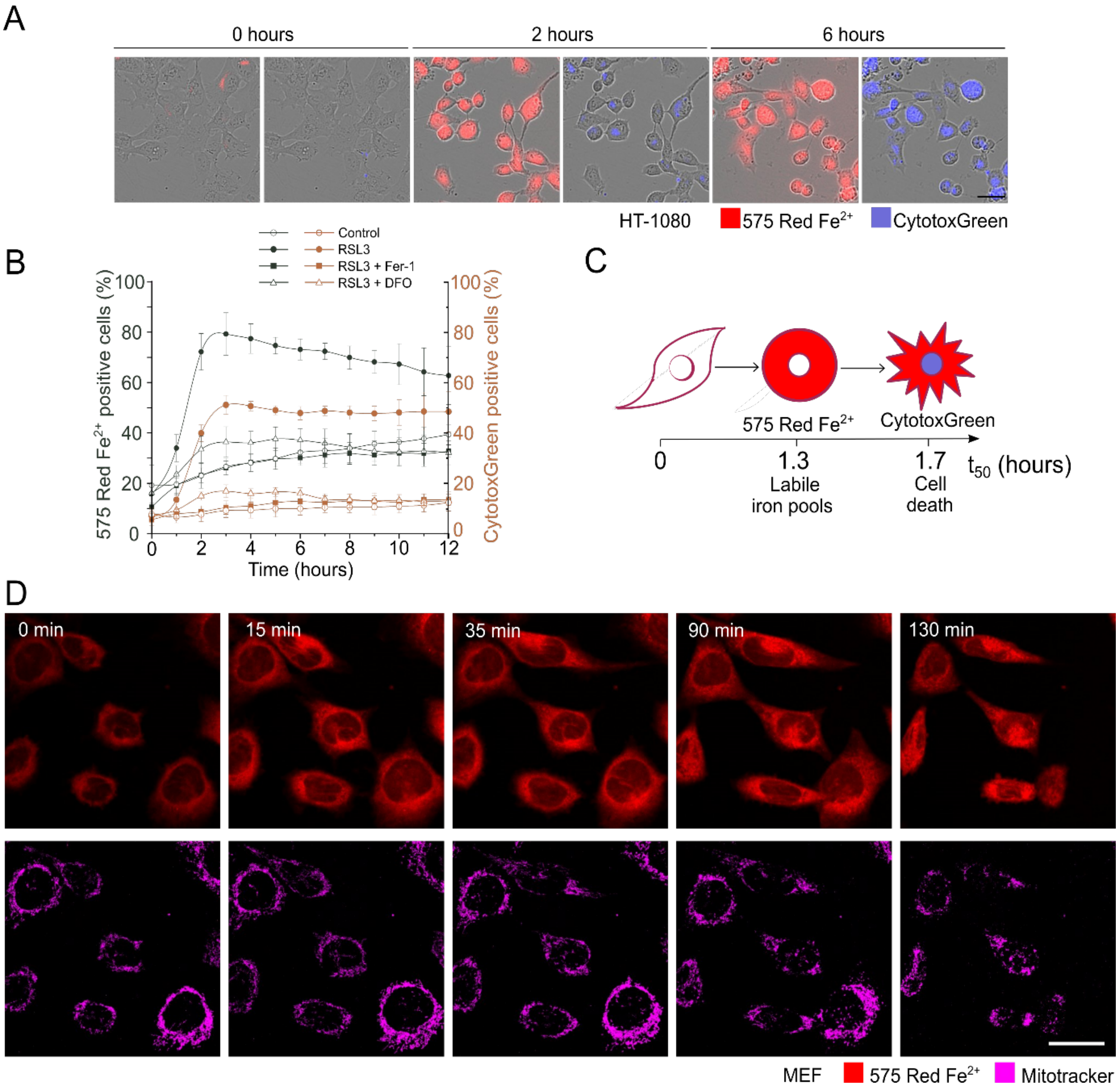

Fig. S1 Increase in labile iron occurs prior to plasma membrane rupture. a Time-lapse Incucyte images of HT-1080 cells treated with RSL3 [0.5 µM] and labeled with Bio Tracker TM 575 Red Fe^{2+} [4 µM] and [1 µM] and CytotoxGreen [250 nM]. Images are representative of 6 replicates from two independent experiments. Scale bar, 50 µm. **b** Kinetics of increase in labile iron pools and cell death after RSL3 [0.5 µM] treatment in the presence or absence of Fer-1 [2 µM] or DFO [10 µM], calculated from experiments as the one shown in **a**. Values represent the mean of 6 replicates made in two independent experiments +/- STDEV. **c** Graphical

representation of the sequence of events observed in HT-1080 cells following RSL3-induced ferroptosis. The t_{50} of each phenotypic event was calculated from the mean curves shown in **b**. These values correspond to the time at 50% of the maximum signal. **d** Time-lapse confocal images of MEF wt cells treated with RSL3 [2 µM] and monitored for Bio Tracker TM 575 Red Fe^{2+} [4 µM] and Mitotracker Deep Red [150 nM] signal. Scale bar, 25 µm.

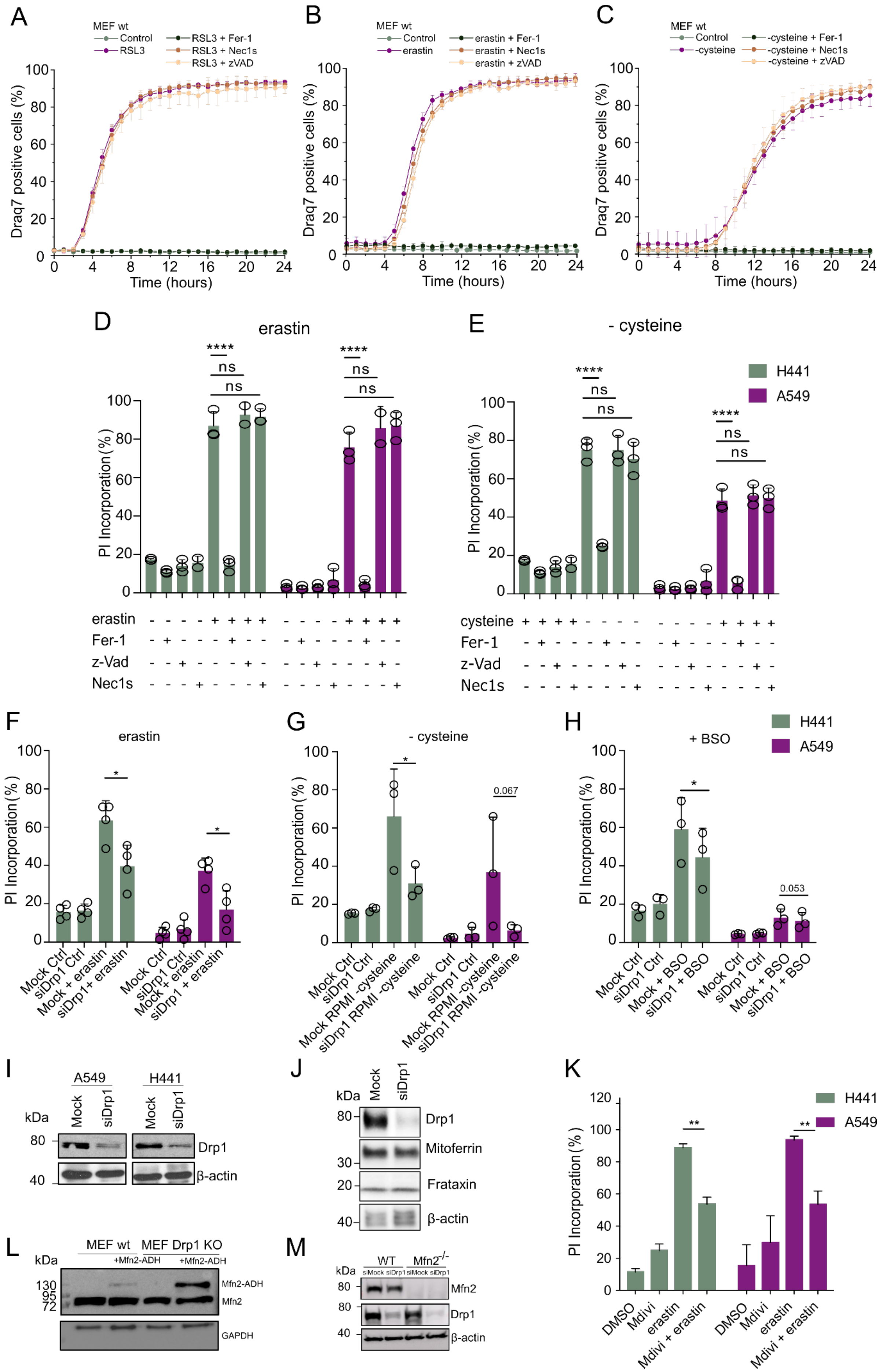

A
B
C
MEF wt
Control
RSL3
RSL3 + Fer-1
RSL3 + Nec1s
RSL3 + zVAD
erastin
erastin + Fer-1
erastin + Nec1s
erastin + zVAD
-cysteine
-cysteine + Fer-1
-cysteine + Nec1s
-cysteine + zVAD
Draq7 positive cells (%)
Time (hours)
D
erastin
E
- cysteine
H441
A549
PI Incorporation (%)
erastin
Fer-1
z-Vad
Nec1s
cysteine
F
G
H
+ BSO
Mock Ctrl
siDrp1 Ctrl
Mock + erastin
siDrp1+ erastin
Mock RPMI -cysteine
siDrp1 RPMI -cysteine
Mock + BSO
siDrp1 + BSO
0.067
0.053
I
J
K
A549
H441
kDa
Mock
siDrp1
Drp1
β-actin
Mitoferrin
Frataxin
DMSO
Mdivi
Mdivi + erastin
L
MEF wt
MEF Drp1 KO
+Mfn2-ADH
Mfn2-ADH
Mfn2
GAPDH
M
WT
Mfn2-/-
siMock
siDrp1

33

34

Fig. S2 Validation of ferroptotic cell death. a-c wild type MEFs were treated with **(a)** RSL3 [0.5 µM], **(b)** Erastin-1 [10 µM] or **(c)** cysteine starvation +/- Fer-1 [2 µM], Nec-1s [10 µM] or zVAD [20 µM] in the presence of Draq7 [3 µM] for the indicated time. Dead cells were quantified as Draq7 positive cells using Incucyte live cell imaging. **d, e** A549 and H441 cells were treated with **(d)** erastin [10 uM] for 24 hours or **(e)** cultured in cystine-depleted medium for 72 hours +/- Fer-1 [2 µM], Nec-1s [10 µM] or zVAD [20 µM]. Cell death was determined by propidium iodide (PI) uptake using flow cytometry. **f-g** A549 or H441 cells were subjected to mock or Drp1-targeting siRNA for 72 hours followed by treatment with (**f**) Erastin-1 [10 µM] for 24 hours or (**g**) cysteine starvation for 72 hours or (**h**) BSO [10 µM] for 48 hours. Cell death was determined as described in **d**. **i** Representative immunoblots of cells using in **d** are shown. **j** Representative immunoblot of mitochondrial iron handling proteins in wild-type MEFs subjected to mock or Drp1-targeting siRNA for 72 hours. **k** A549 and H441 cells were pretreated Mdivi-1 [75 µM] for 48 hours followed by treatment with erastin [10 µM] for 24 hours. **l** Representative Western blots showing the expression of Mfn2-ADH in control or Drp1 knockout (KO) MEFs. **m** Representative immunoblots of cells as shown in **Fig 2 j.** Data represent means of three independent experiments +/- STDEV. Two-way ANOVA and Tukey's multiple comparison test, ****$p < 0.0001$, ***$p < 0.001$, **$p < 0.01$, *$p < 0.05$.

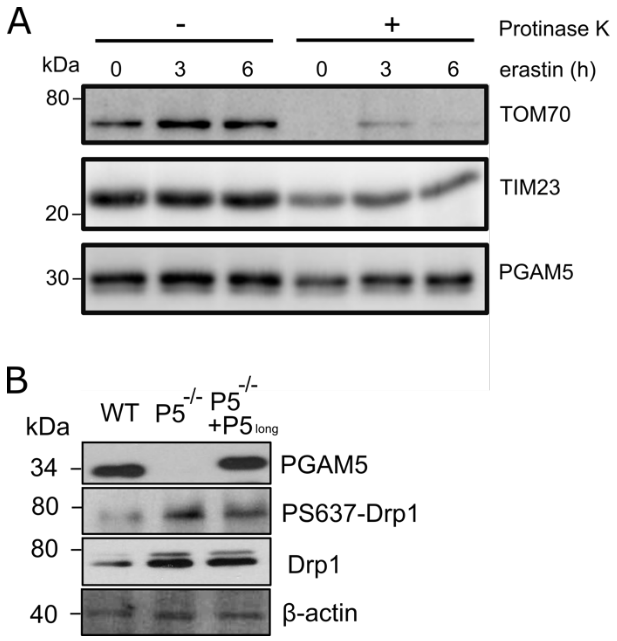

Fig. S3 Ferroptosis does not alter PGAM5 IMM/OMM localization. **a** Representative immunoblot of A549 treated with erastin [10 µM] for 0, 3 or 6 hours (h) and subsequent mitochondrial isolation. Mitochondrial outer membrane proteins were digested using Proteinase K [0.5 µg/mL] for 10 min. **b** Representative immunoblot of Drp1 phosphorylation at S367 in wild type and PGAM5-/- HEK cells or PGAM5-/- HEK cells containing a doxycycline-inducible PGAM5-expression plasmid. Data are representative of at least three independent experiments.
