# Supplementary material for: Ferroptosis triggers mitochondrial fragmentation via Drp1 activation
Source: Cell Death Dis. 2025 Jan 25;16(1):40. doi: 10.1038/s41419-024-07312-2 (PMC11762985; doi:10.1038/s41419-024-07312-2)
Supplement: Supplementary file 2 — Original Data File [file 41419_2024_7312_MOESM2_ESM.docx]

**Full and uncropped Western Blots:**

**
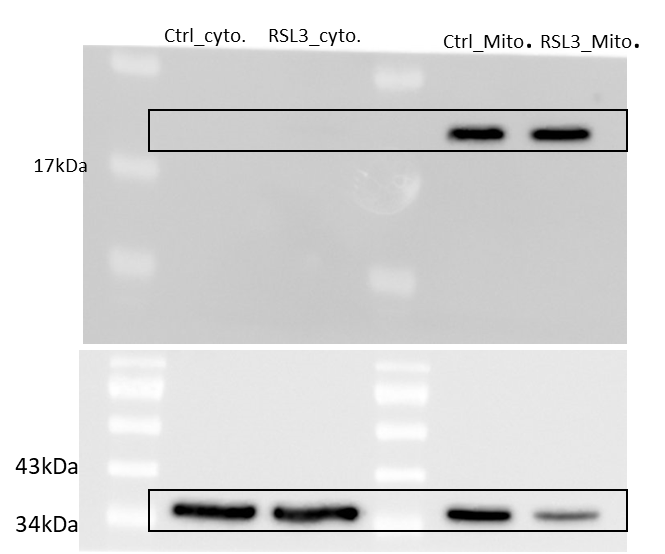
**

**Smac release and GAPDH – Figure 1I (upper panel)**


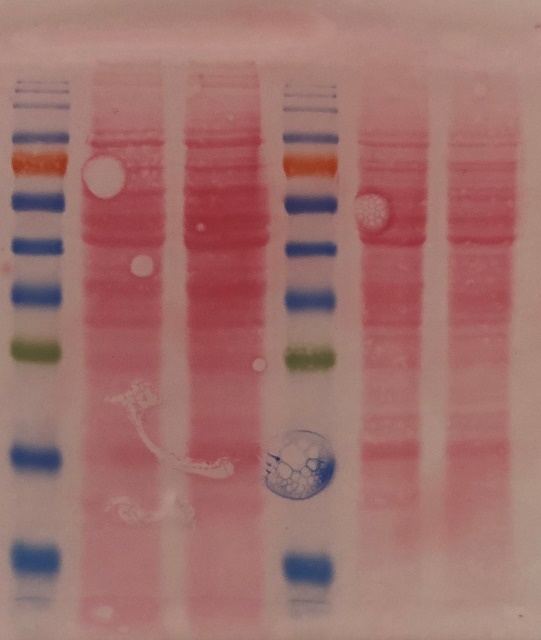


**Smac release – Figure 1I (upper panel, corresponding Ponceau)**

**
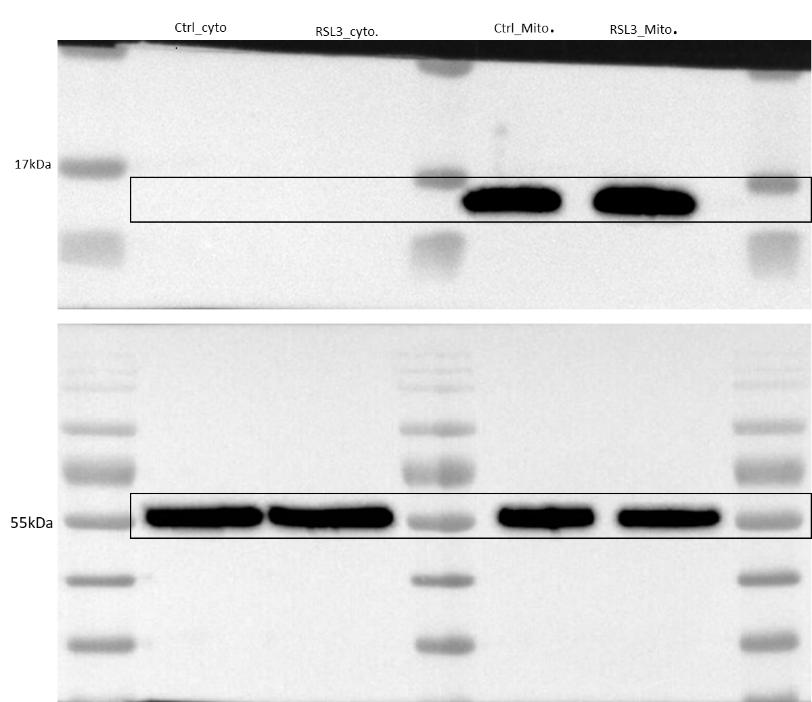
**

**Cytochrome C release and β tubulin– Figure 1I (lower panel)**

**
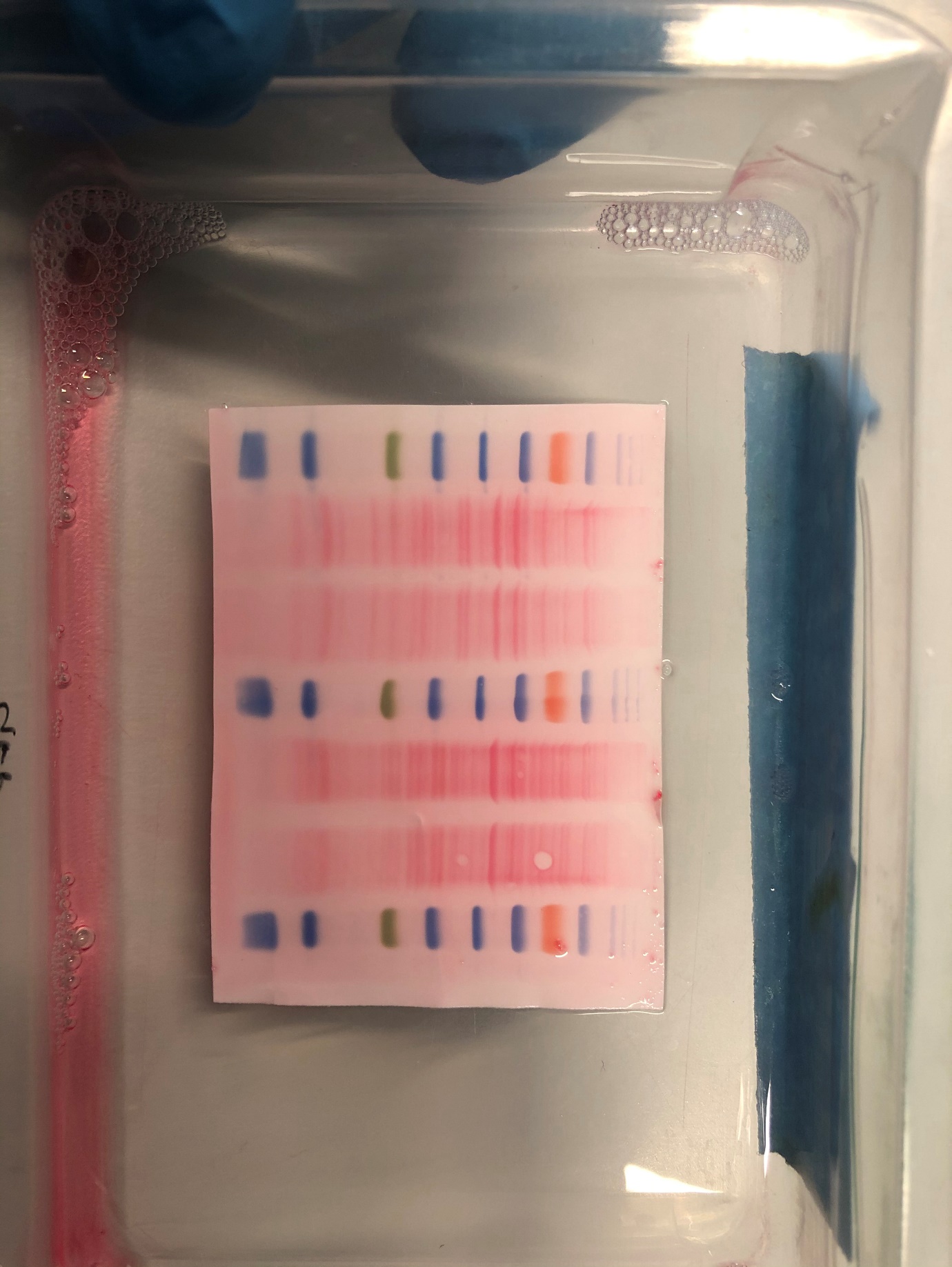
**

**Cytochrome C release – Figure 1I (lower panel, corresponding Ponceau)**


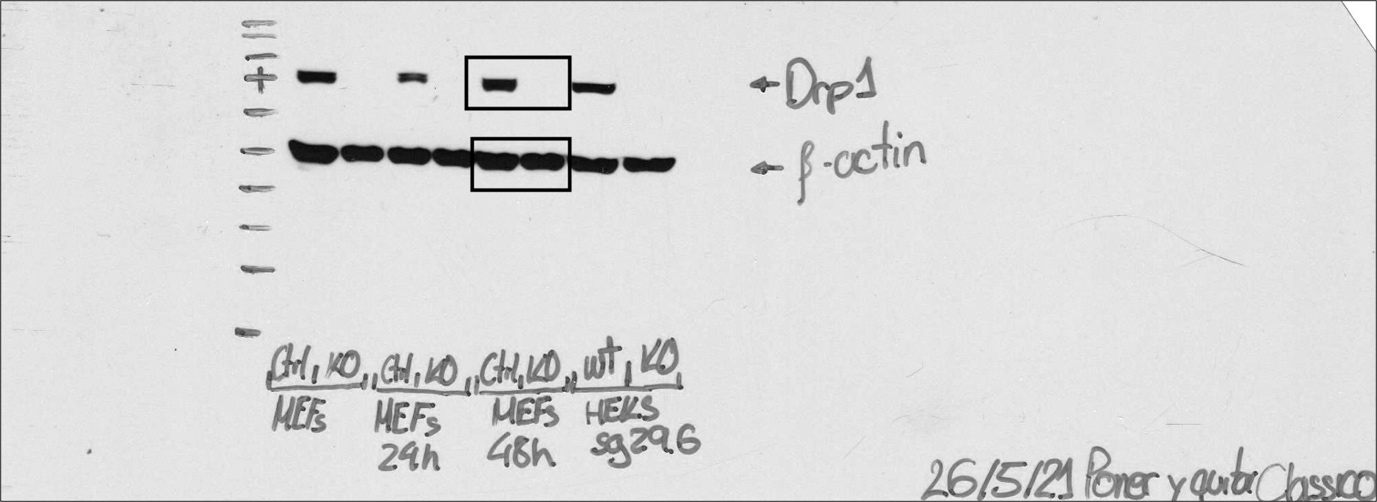


**Figure 2D**


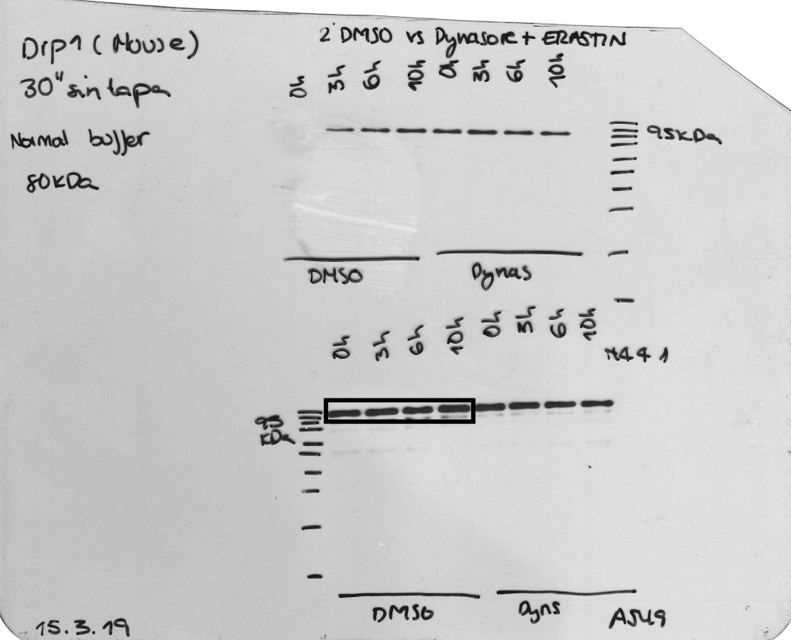

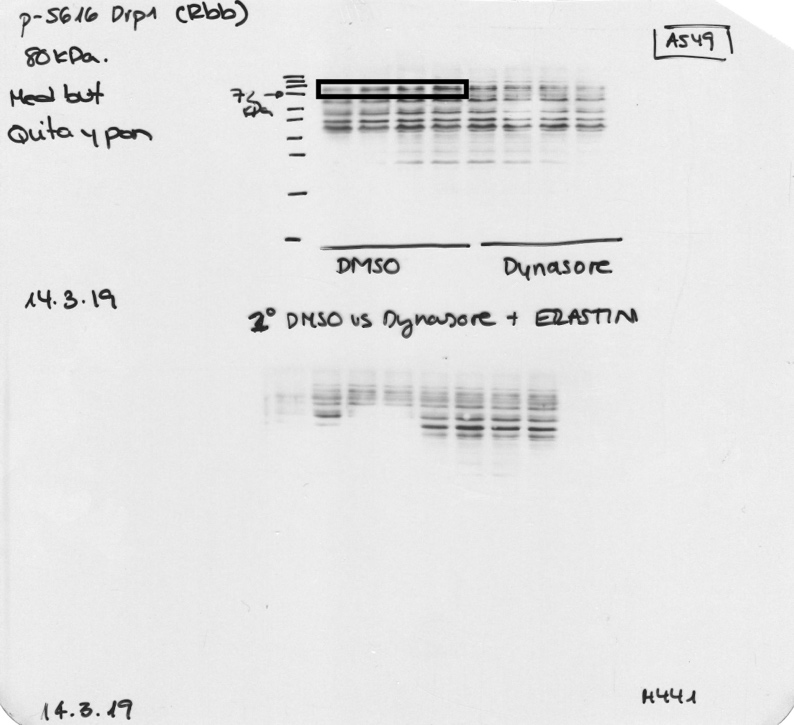

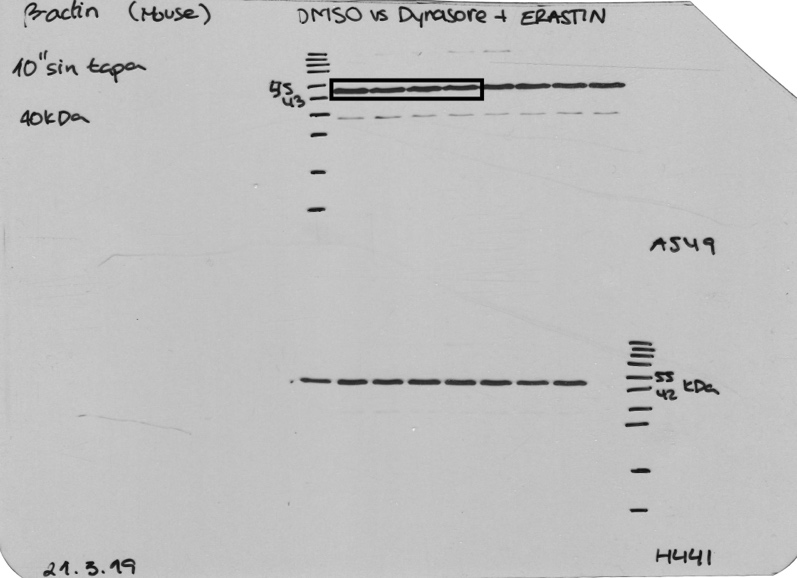


**Figure 3A**


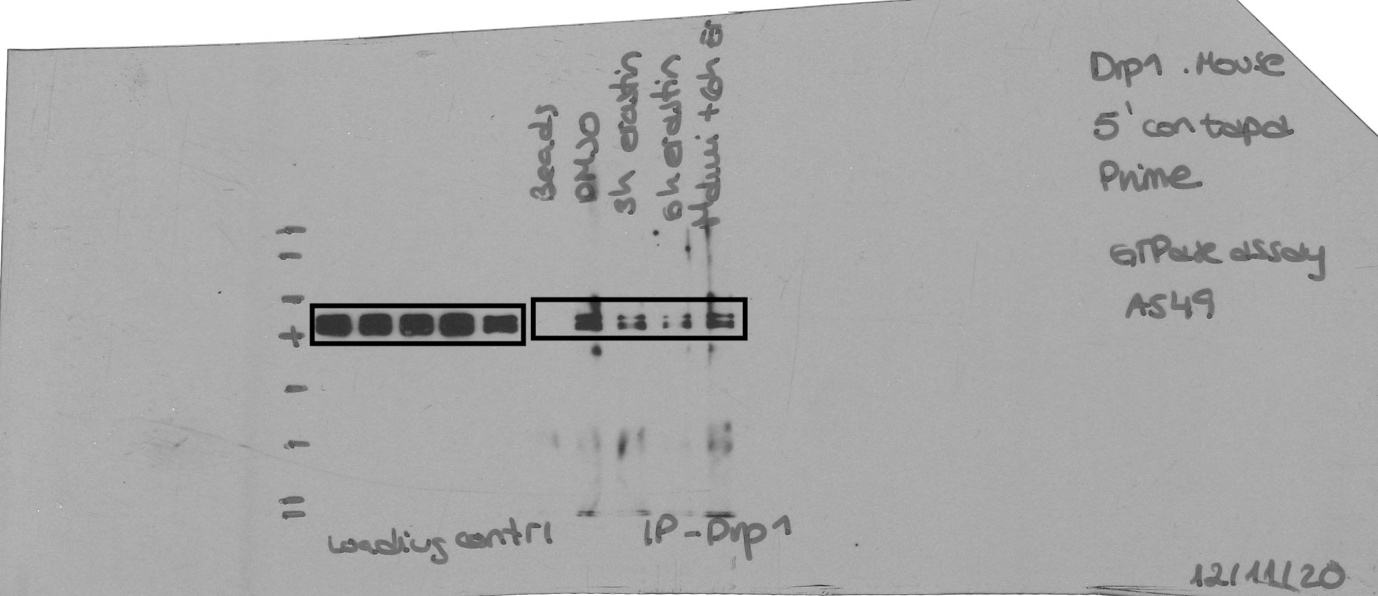


**Figure 3D**


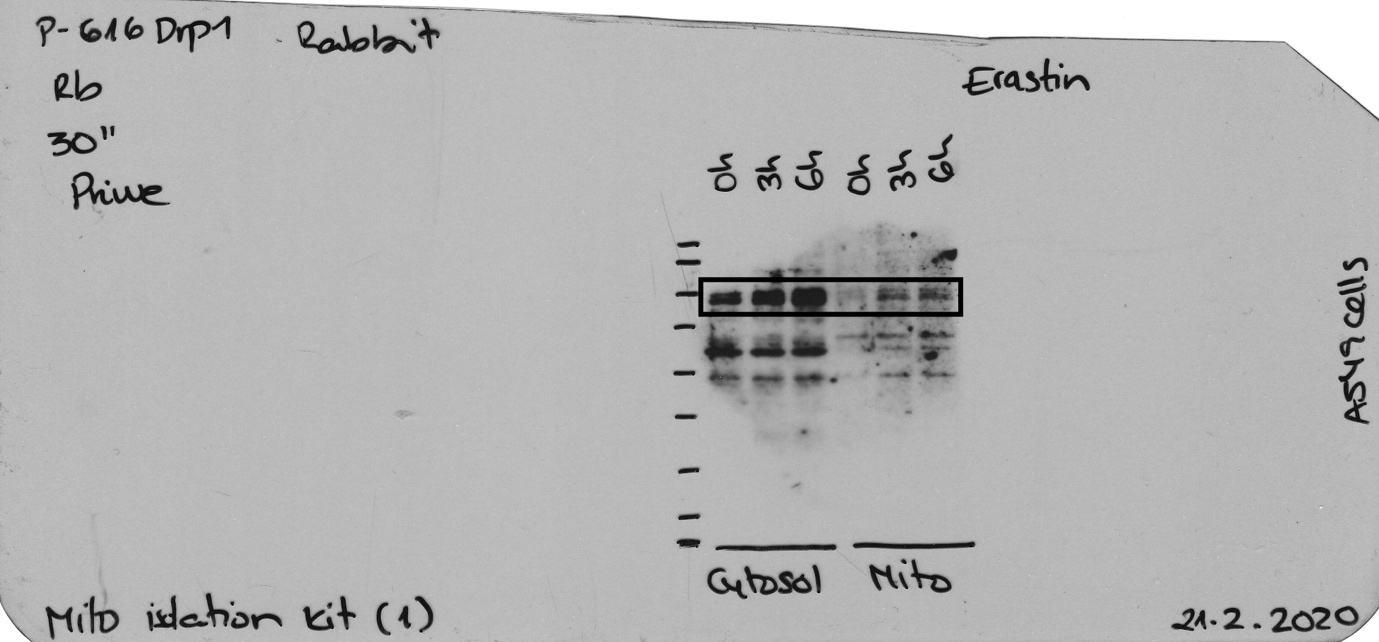

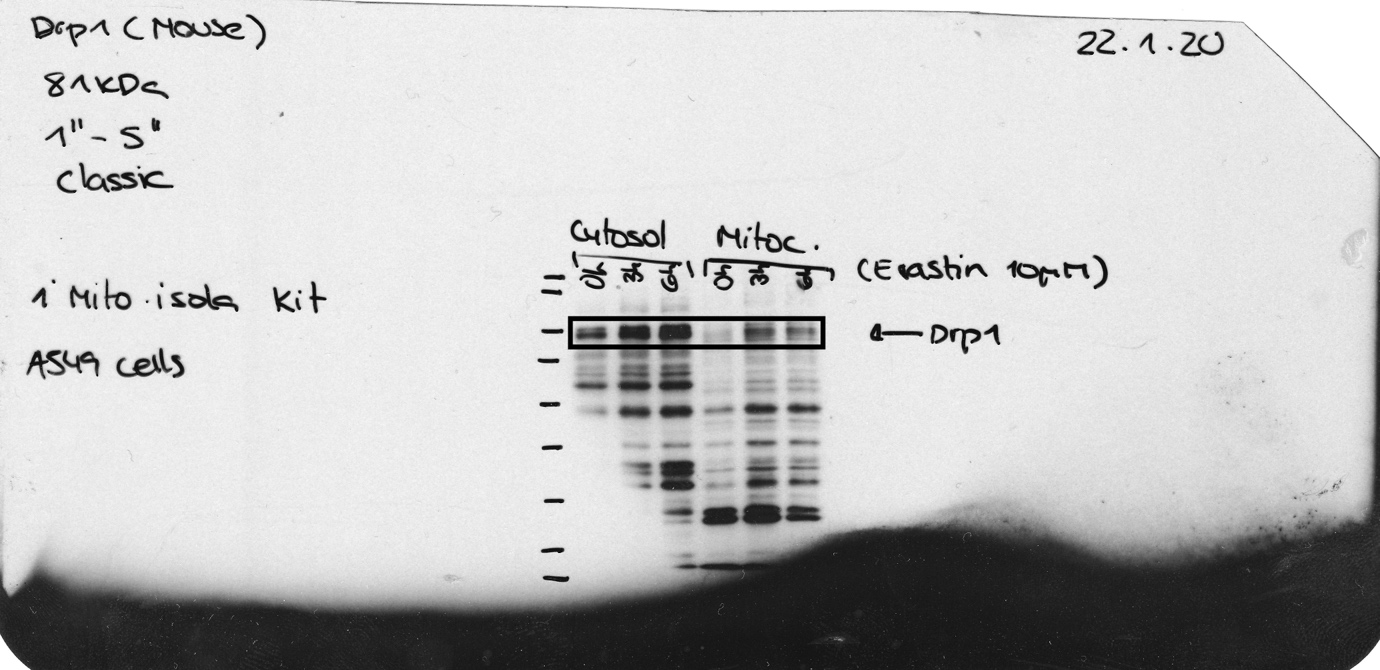

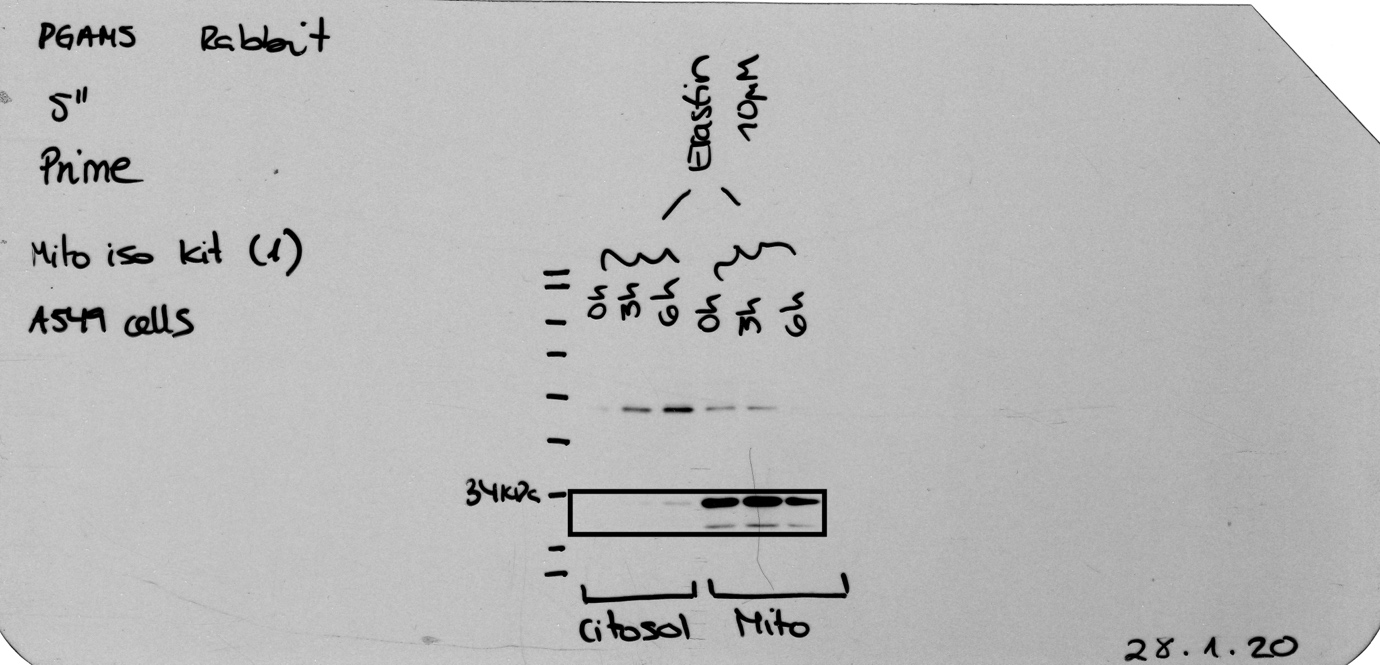


**Figure 3G**


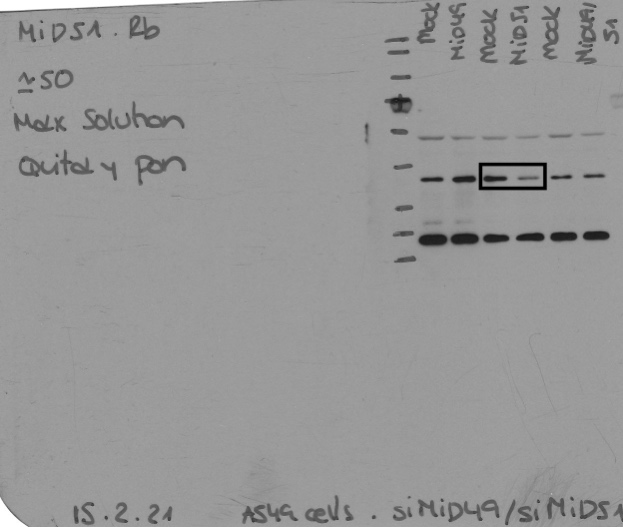

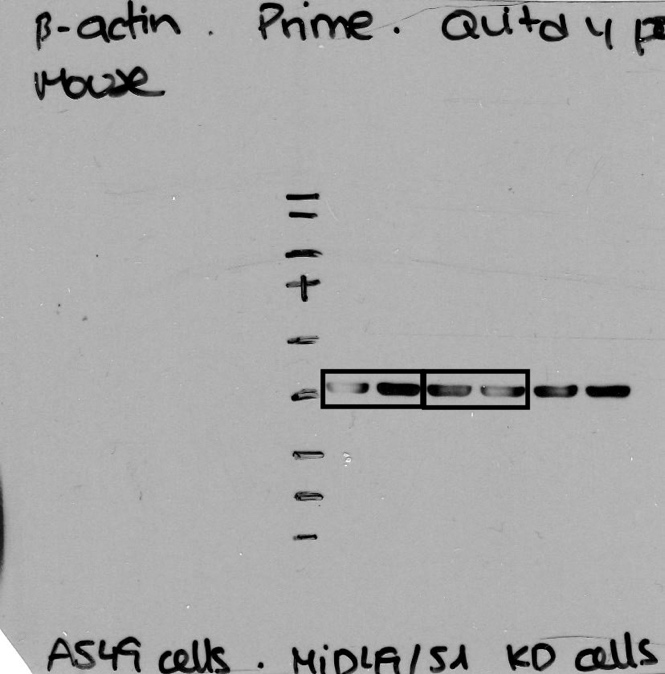

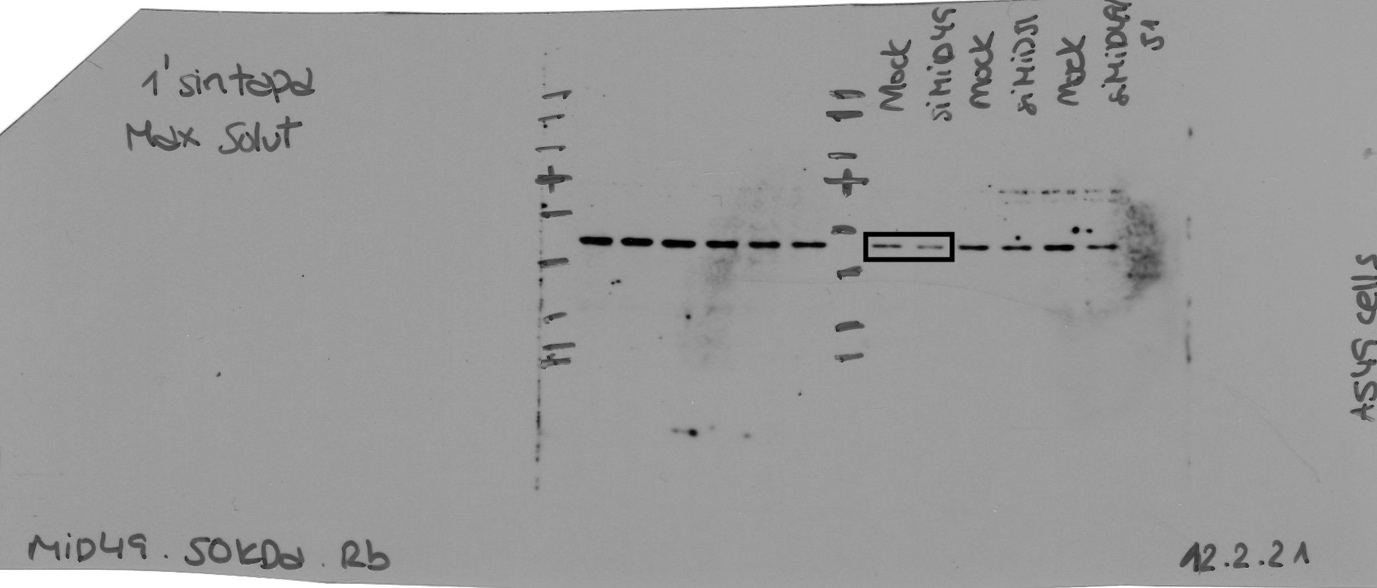


**Figure 4B and C**


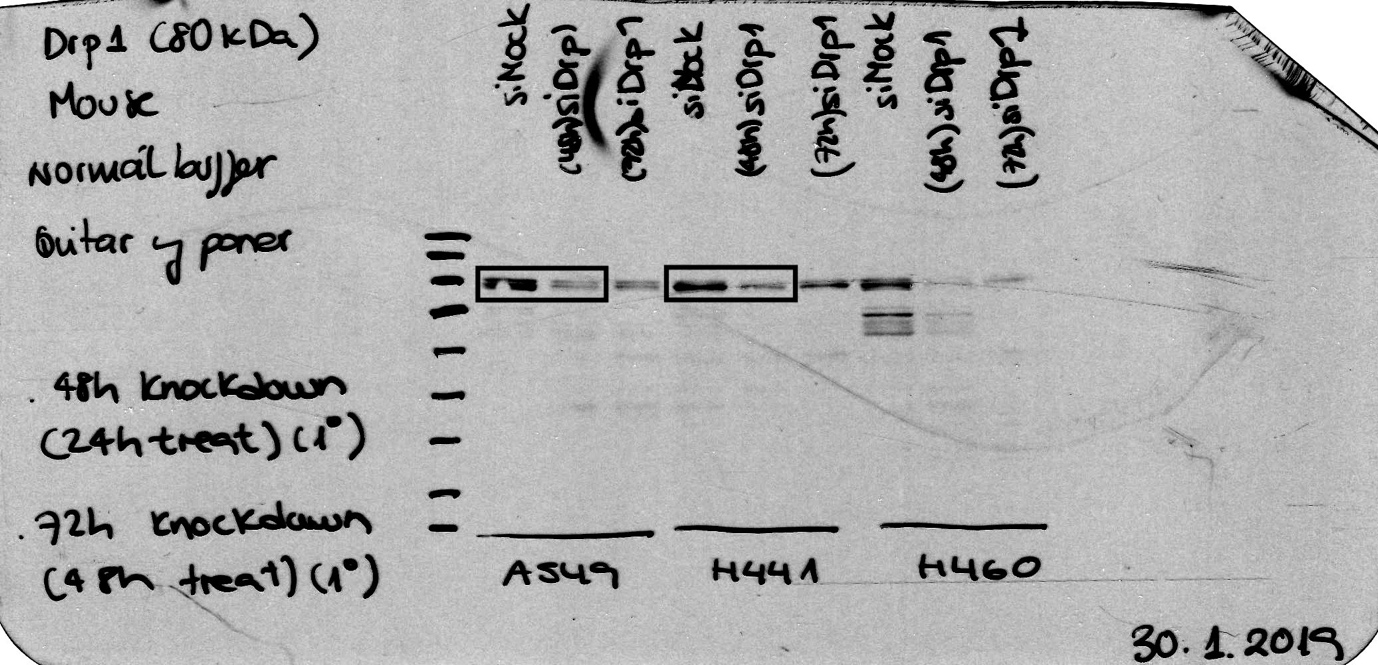

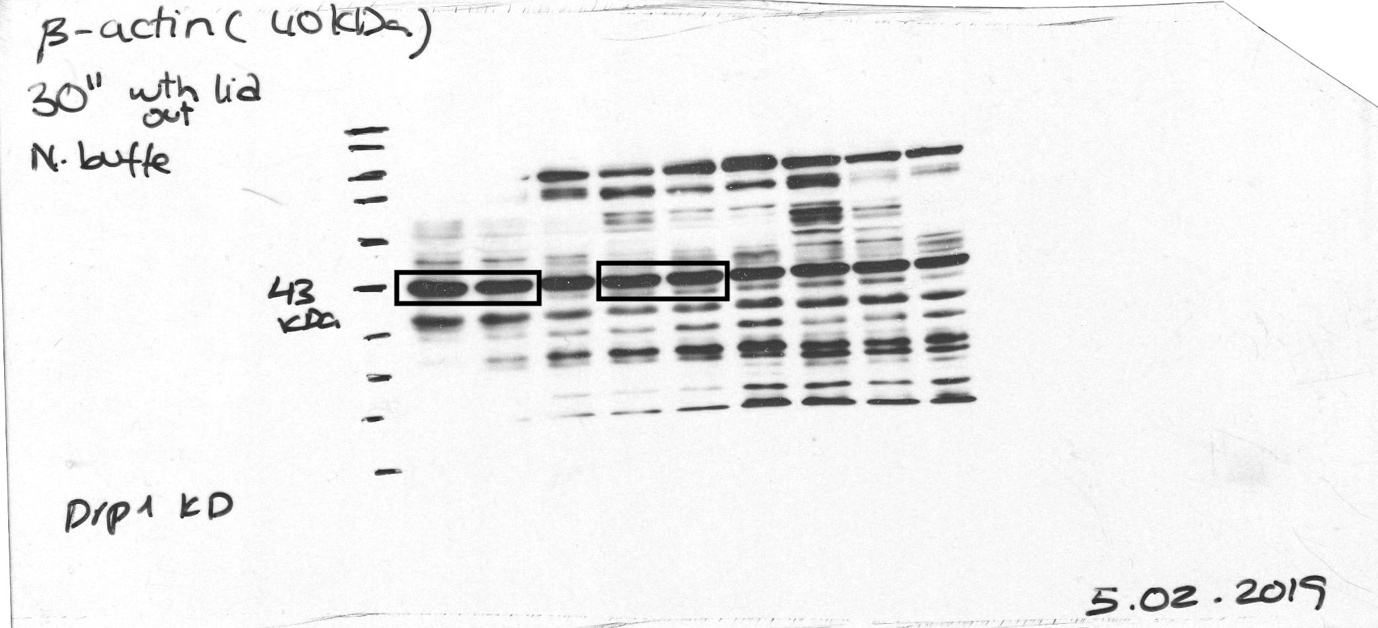


**Figure S2I**

**Figure S2J**

**
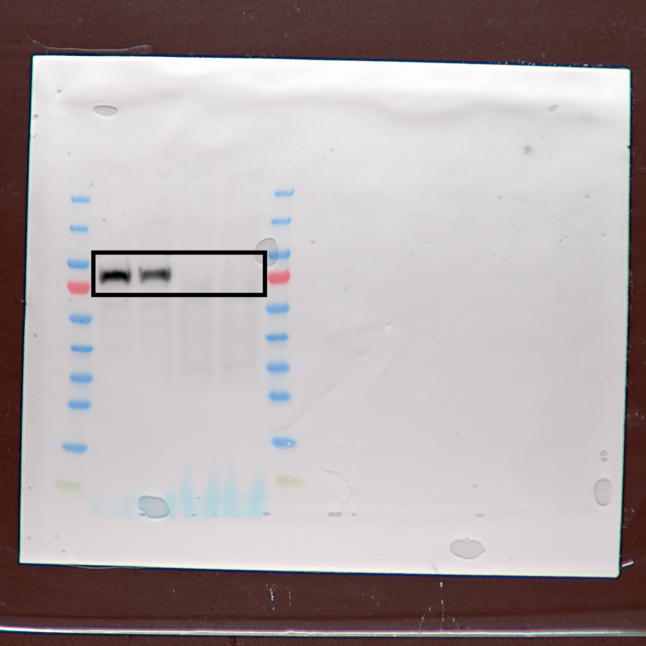
**

**Mitofusin**

**
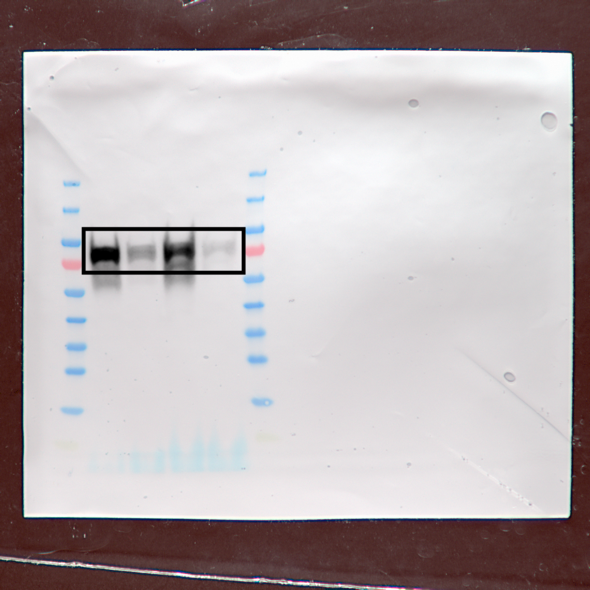
**

**Drp1**

**
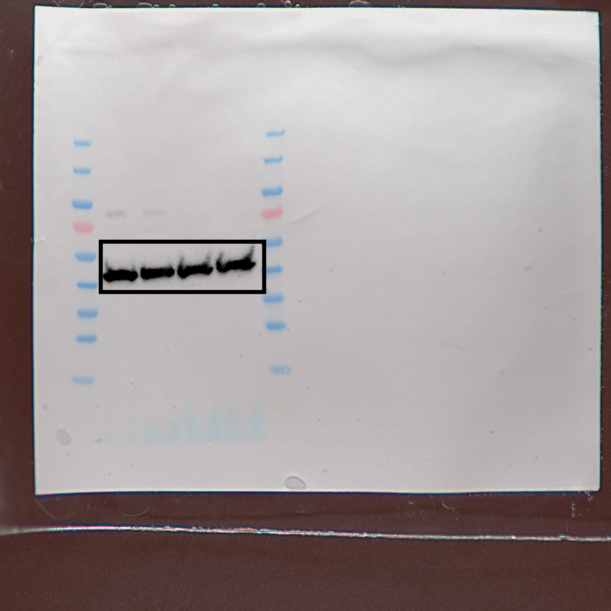
**

**Actin**

**Figure S2M**

**PGAM5 – Figure S3A**

**TIM23– Figure S3A**

**TOM70– Figure S3A**

**
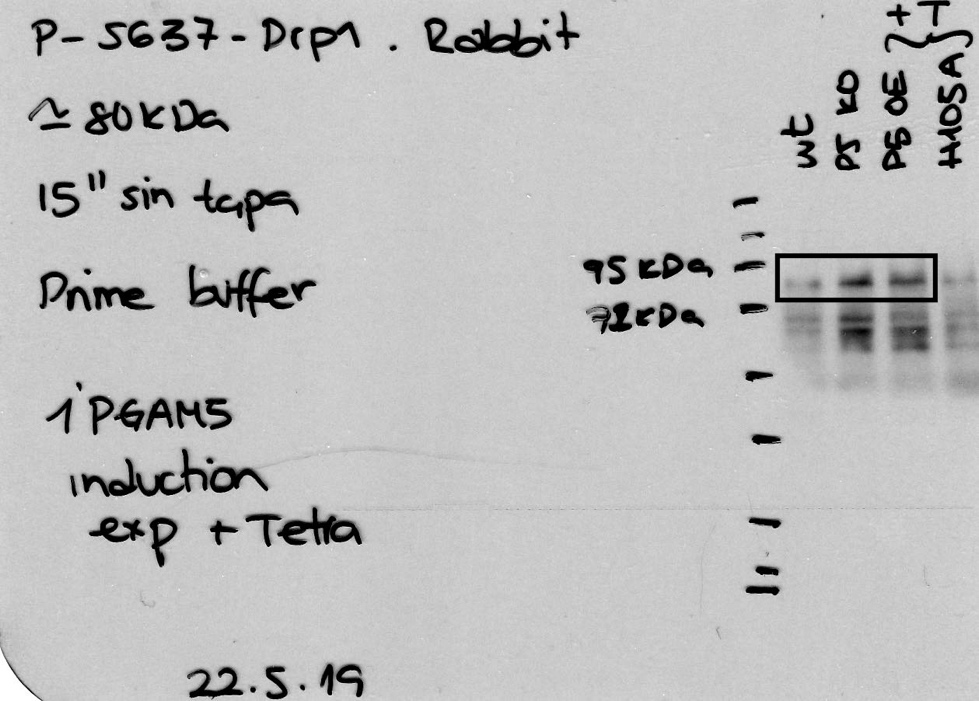

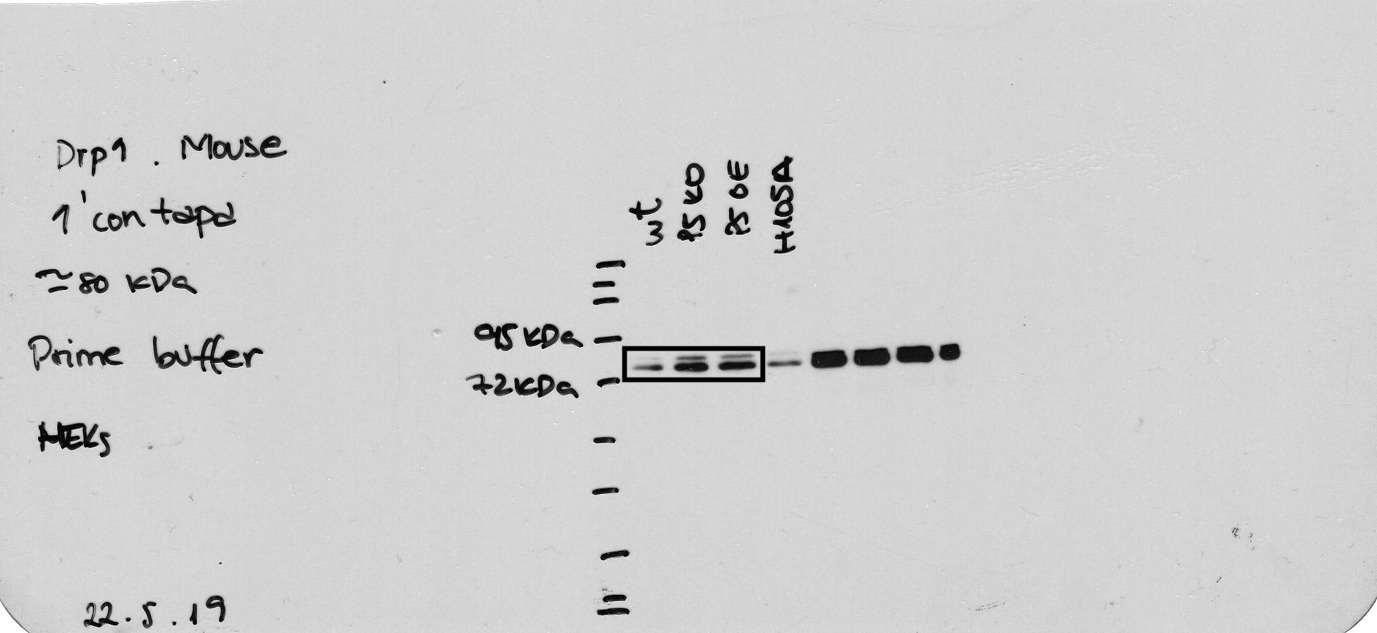

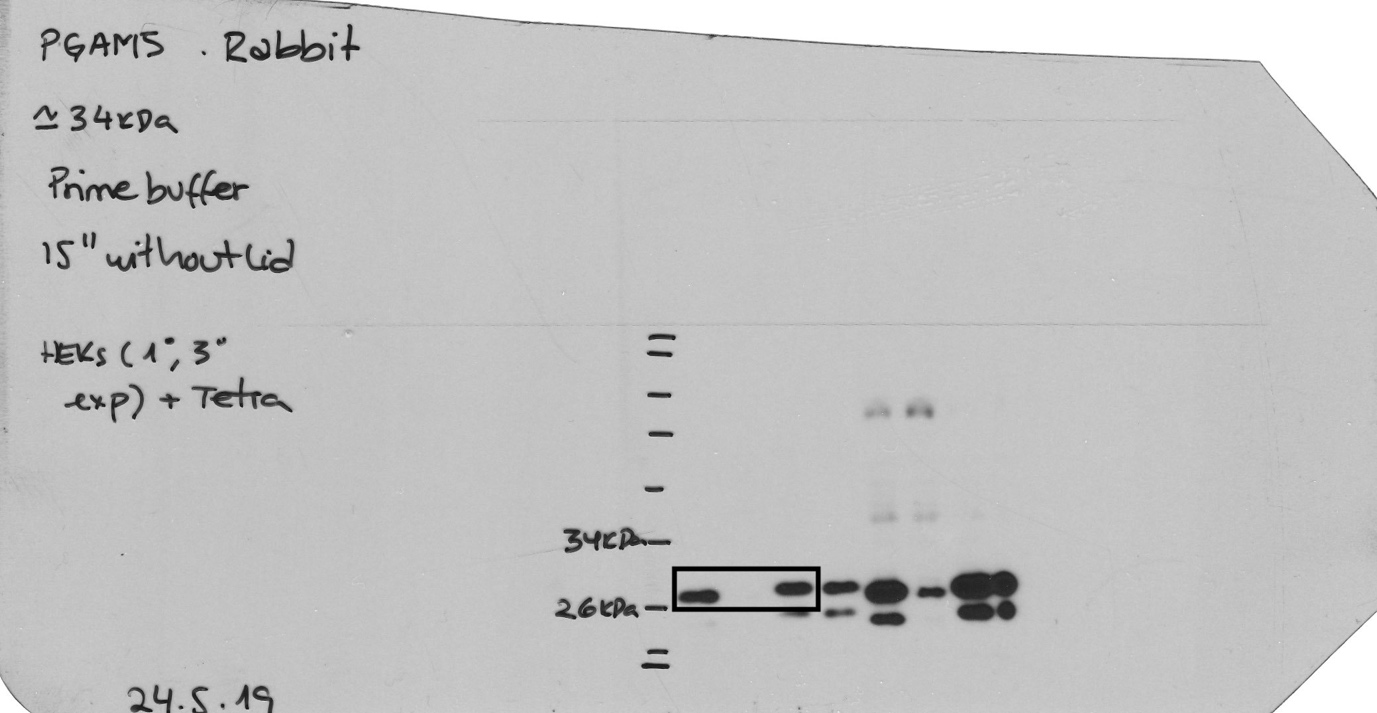

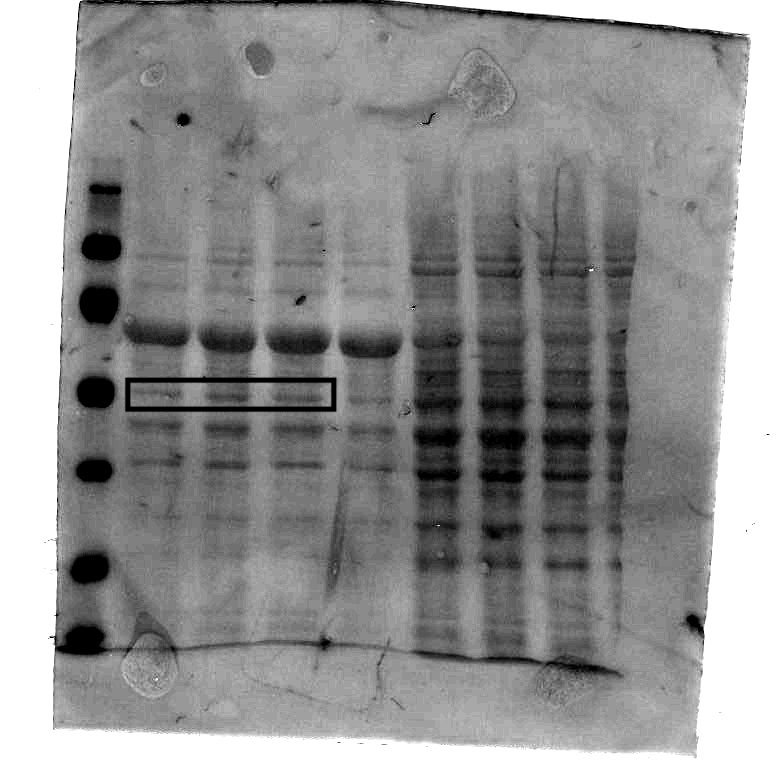
beta-Actin**

**Figure S3B**
